# Terpene synthases and their contribution to herbivore-induced volatile emission in western balsam poplar (*Populus trichocarpa*)

**DOI:** 10.1186/s12870-014-0270-y

**Published:** 2014-10-11

**Authors:** Sandra Irmisch, Yifan Jiang, Feng Chen, Jonathan Gershenzon, Tobias G Köllner

**Affiliations:** Max Planck Institute for Chemical Ecology, Hans-Knöll-Strasse 8, D-07745, Jena, Germany; Department of Plant Sciences, University of Tennessee, Knoxville, TN37996, USA

**Keywords:** *Populus trichocarpa*, Sesquiterpenes, Monoterpenes, Volatiles, Terpene synthase gene family, Jasmonic acid

## Abstract

**Background:**

As a response to caterpillar feeding, poplar releases a complex mixture of volatiles which comprises several classes of compounds. Poplar volatiles have been reported to function as signals in plant-insect interactions and intra- and inter-plant communication. Although the volatile blend is dominated by mono- and sesquiterpenes, there is much to be learned about their formation in poplar.

**Results:**

Here we report the terpene synthase (TPS) gene family of western balsam poplar (*Populus trichocarpa*) consisting of 38 members. Eleven *TPS* genes (*PtTPS5*-*15*) could be isolated from gypsy moth (*Lymantria dispar*)-damaged *P. trichocarpa* leaves and heterologous expression in *Escherichia coli* revealed TPS activity for ten of the encoded enzymes. Analysis of *TPS* transcript abundance in herbivore-damaged leaves and undamaged control leaves showed that seven of the genes, *PtTPS6*, *PtTPS7*, *PtTPS9*, *PtTPS10*, *PtTPS12*, *PtTPS13* and *PtTPS15*, were significantly upregulated after herbivory. Gypsy moth-feeding on individual leaves of *P. trichocarpa* trees resulted in induced volatile emission from damaged leaves, but not from undamaged adjacent leaves. Moreover, the concentration of jasmonic acid and its isoleucine conjugates as well as *PtTPS6* gene expression were exclusively increased in the damaged leaves, suggesting that no systemic induction occurred within the tree.

**Conclusions:**

Our data indicate that the formation of herbivore-induced volatile terpenes in *P. trichocarpa* is mainly regulated by transcript accumulation of multiple *TPS* genes and is likely mediated by jasmonates. The specific local emission of volatiles from herbivore-damaged leaves might help herbivore enemies to find their hosts or prey in the tree canopy.

**Electronic supplementary material:**

The online version of this article (doi:10.1186/s12870-014-0270-y) contains supplementary material, which is available to authorized users.

## Background

Volatile organic compounds (VOCs) play multiple roles in the interactions of plants with their environment. Floral and fruit VOCs, for example, are known as attractants for pollinators and seed dispersers, respectively, while vegetative VOCs are reported to have various functions in inter- and intra-plant communication and plant defense against herbivores and pathogens [1,2]. The emission of VOCs from vegetative plant organs is often induced by biotic stresses like insect herbivory [3,4]. Such induced volatile blends can attract natural enemies of the herbivores, a reaction termed indirect defense [2]. For example, the volatile blends from herbivore-infested *Arabidopsis thaliana*, black poplar (*Populus nigra*) and maize (*Zea mays*) have been described to be attractive for different parasitoids [5-7]. However, beside their role as signals in indirect defense, herbivore-induced vegetative VOCs can also function in direct defense as toxins and repellants for herbivores [8-10].

In general, herbivore-induced volatile blends are often dominated by terpenes but also comprise other classes of natural compounds including green leaf volatiles, alcohols, esters, and nitrogen-containing volatiles. Terpenes represent the largest and most diverse group of plant secondary metabolites [1]. They are built up of isoprenoid (C_5_) units which have their origin either in the mevalonate pathway or in the 2-C-methylerythritol-4-phosphate (MEP) pathway. A head to tail condensation of such C_5_ units catalyzed by prenyltransferases leads to the formation of geranyl diphosphate (GPP), farnesyl diphosphate (FPP), and geranylgeranyl diphosphate (GGPP). Terpene synthases (TPSs), the key enzymes of terpene metabolism, convert these precursors into the huge number of different terpene carbon skeletons [11]. Most plant genomes possess a mid-size gene family encoding terpene synthases [12]. Based on their phylogenetic relationships, plant TPSs can be classified into seven different clades [12,13]. TPS-a, TPS-b and TPS-g are angiosperm-specific clades with the TPS-a clade containing predominantly sesquiterpene synthases and the TPS-b and TPS-g clades consisting mostly of monoterpene synthases. The TPS-d clade comprising mono-, sesqui- and diterpene synthases and the TPS-h clade comprising diterpene synthases are gymnosperm- and lycopod- (*Selaginella moellendorfii*) specific, respectively. The gymnosperm and angiosperm copalyl diphosphate synthases (CPS) and kaurene synthases (KS) make up the TPS-c and TPSe/f clades, respectively. Recently, a new class of terpene synthases was found in *S. moellendorffii* which showed sequence similarity to microbial terpene synthases and were designated as microbial terpene synthase like (MTPSL) genes [14].

While a few terpene synthases do function in plant primary metabolism, for example, in gibberellin biosynthesis, the majority functions in the biosynthesis of secondary metabolites involved in ecological interactions. Due to the prominent occurrence of terpenes in herbivore-induced volatile blends [3,9,15], terpene synthases have received a lot of attention and much evidence exists for their involvement in plant defense. For example, the introduction of a sesquiterpene synthase gene from wild tomato (*Solanum habrochaites*) into a cultivated tomato line resulted in an increased herbivore resistance [16]. The overexpression of a linalool synthase in *A. thaliana* also increased the resistance of this plant against aphids [17]. Additionally, herbivory-induced terpene synthases from lima bean (*Phaseolus lunatus*) and maize, for example, are reported to produce sesquiterpenes which have been shown to attract natural enemies of insect herbivores [18,19].

The majority of information about plant responses to herbivory is mainly based on herbaceous plants. However, in recent years an increasing number of studies on volatile-mediated plant defense have been carried out on woody species from both the gymnosperms and angiosperms. It is known, for example, that poplar emits a complex volatile blend after being damaged by herbivores [7,10,20,21]. Although monoterpenes and sesquiterpenes are described to be the most dominant compounds emitted, only 8 terpene synthase genes have been isolated and characterized from poplar to date. These include the isoprene synthase (IPS) from *P. alba* x *P. tremula* [22], PtdTPS1 from *P. trichocarpa* x *P. deltoides* [20], PnTPS1 and PnTPS2 from black poplar (*P. nigra*) [7] and four TPS (PtTPS1-4) from western balsam poplar (*P. trichocarpa*) [21], the species of *Populus* that has been fully sequenced [23].

In this report we describe the identification and functional characterization of the *TPS* gene family of *P. trichocarpa* consisting of thirty-eight members. Fifteen *TPS* genes could be isolated from herbivore-damaged leaves, of which eleven have not been characterized before. A qRT-PCR analysis revealed that the majority of these genes were upregulated after herbivory indicating their potential involvement in plant defense. To study the spatial regulation of herbivore-induced volatile biosynthesis in more detail, we carried out a comprehensive volatile collection from a single herbivore-infested leaf and from individual neighboring undamaged leaves and compared the observed volatile pattern with *TPS* gene expression data as well as with phytohormone levels in these tissues.

## Results

### The *TPS* gene family in *P. trichocarpa*

**Figure 1:**
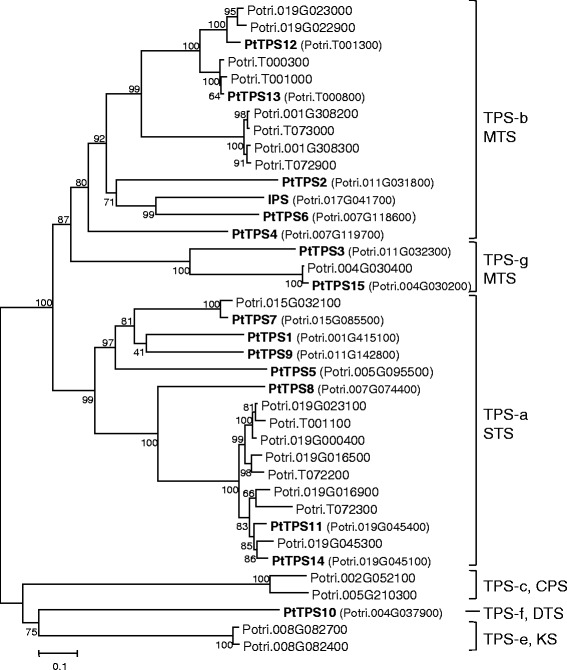
**Phylogenetic tree of full**-**length terpene synthases** **(TPS)** **from** ***Populus trichocarpa*** **.** The phylogenetic relationship of 38 *P. trichocarpa* TPS is shown. PtTPS1-4 and IPS have been characterized in previous studies. The tree was inferred with the neighbor-joining method and n = 1000 replicates for bootstrapping. Bootstrap values are shown next to each node. IPS, isoprene synthase; STS, sesquiterpene synthase; MTS, monoterpene synthase; CDS, copalyl diphosphate synthase; DTS, diterpene synthase; KS, kaurene synthase. TPS-a to h represent TPS subfamilies.

To identify the members of the *TPS* gene family in *P. trichocarpa*, we conducted a BLAST analysis using the second improved version of the poplar genome (v3 assembly, http://www.phytozome.net/poplar). This analysis revealed 38 full length *TPS* genes which encode for putative proteins with a minimal length of 520 amino acids including the previously published genes *PtTPS1*-*4* [21] (Figure 1). Additionally, 19 *TPS* gene fragments were found in the database. Bacterial-like *TPSs* as already described for *S. moellendorffii* [14] were not identified in the poplar genome. Six of the seven *TPS* gene subfamilies were represented in the 38 complete poplar *TPSs* (Additional file 1: Figure S1) [12]. The *TPS*-*a* subfamily with 16 members and the *TPS*-*b* subfamily with 17 members made up the majority of poplar *TPSs*, while only two members each fell into the *TPS*-*g*, *TPS*-*c* and the *TPS*-*e* subfamilies and only one *TPS* gene clustered within the *TPS*-*f* subfamily. As the members of the *TPS*-*c* and *TPS*-*e* subfamily most likely represent copalyl diphosphate synthases and kaurene synthases (for conserved protein sequence motifs see Additional file 1: Figure S2), respectively, which are not involved in volatile biosynthesis, we did not focus on these in more detail in this study. The chromosomal position was assigned to 29 of the full-length *TPS* genes, and these were found to be located on eleven of the nineteen poplar chromosomes. About one third of the *TPS* genes and half of the *TPS* gene fragments were found on chromosome 19, suggesting the occurrence of multiple duplication and recombination events on this chromosome (Additional file 1: Figure S3). On other chromosomes, a maximum number of three *TPS* genes were found. Nine *TPS* genes and 4 *TPS* gene fragments were not linked to poplar chromosomes based on the new genome version. Many of these sequences share a high degree of nucleotide identity which makes annotation and assignment in the genome difficult. Therefore one could still expect changes in the actual numbers of poplar *TPS* genes and their locations as newer versions of the poplar genome are released.

### Isolation of poplar *TPS* genes and their structural features

Using cDNA made from gypsy moth (*Lymantria dispar*)-damaged *P. trichocarpa* leaves, 15 open reading frames of *TPS* genes could be amplified and cloned. Eleven of them represented poplar *TPS* genes which have not been characterized and described before. Following the nomenclature of Danner and coworkers (2011), the genes were designated as *PtTPS5* to *PtTPS15* (Figure 1). Based on their sequence similarity to so far characterized poplar TPS and representative TPS from other plant species (Additional file 1: Figure S4), the proteins encoded by *PtTPS5*-*15* were tentatively classified as four monoterpene synthases (MTS) (PtTPS6, PtTPS12, PtTPS13, PtTPS15), six sesquiterpene synthases (STS) (PtTPS5, PtTPS7, PtTPS8, PtTPS9, PtTPS11, PtTPS14) and a diterpene synthase (DTS) (PtTPS10). PtTPS5-15 all had a length between 550 to 840 amino acids (Additional file 2: Table S1) and contained typical conserved elements including the DDxxD motif and the NSE/DTE motifs (Additional file 1: Figure S5), both of which are involved in the binding of the metal cofactor [24]. Interestingly, PtTPS11 and PtTPS14 possessed an altered NSE/DTE motif which contained a glycine residue instead of the serine/threonine. Another typical sequence motif, the RR (x)_8_ W motif in the N-terminal part was also changed and expressed as RP (x)_8_ W in PtTPS11 and PtTPS14 and was completely absent in PtTPS15. Another conserved protein sequence of terpene synthases is the RxR-motif implicated in the complexation of the diphosphate group after ionization of the substrate [24]. In PtTPS15, the RxR motif was modified to RxQ, which is also present in PtTPS3 [21]. TPS10, a putative diterpene synthase, showed an RxK motif at this position (Additional file 1: Figure S5, Additional file 2: Table S1).

In general, MTS and DTS contain N-terminal signal peptides which target these proteins to the plastids, the site of GPP and GGPP biosynthesis [13]. In contrast, sesquiterpene synthases are localized in the cytosol where FPP serves as the substrate for this enzyme class. The TargetP 1.1 server (http://www.cbs.dtu.dk/services/TargetP/) was used for signal peptide prediction. A plastid transit peptide was predicted for PtTPS6, PtTPS12 and PtTPS13 (Additional file 2: Table S1) supporting their roles as MTS in plastids. However, no signal peptide could be predicted for the putative MTS PtTPS15 and the putative DTS PtTPS10.

### Heterologous expression and *in vitro* functional characterization of poplar TPSs

For functional characterization of poplar TPS, all isolated *TPS* genes were heterologously expressed in *Escherichia coli*. To ensure that the predicted signal peptides did not interfere with expression, truncated versions of *PtTPS6* (Δ57nt), *PtTPS12* (Δ126nt) and *PtTPS13* (Δ126nt) were expressed. The truncations were chosen so that the RR(x) _8_W-motif was still present. Proteins from raw extracts were assayed with GPP, FPP and GGPP, each in the presence of the co-substrate magnesium chloride, to determine monoterpene-, sesquiterpene- and diterpene- forming activity, respectively.

**Figure 2:**
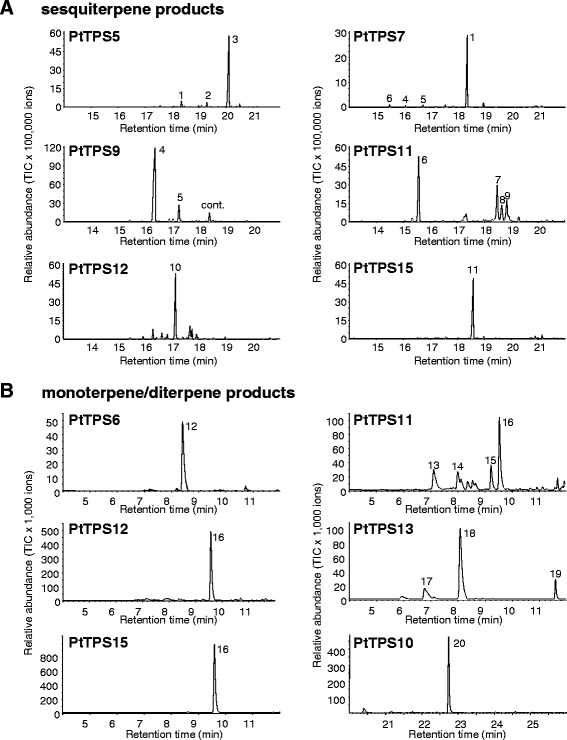
**GC-** **MS analysis of sesquiterpenes** **(A),** **monoterpenes and diterpenes** **(B)** **produced by recombinant PtTPS5,** **PtTPS6,** **PtTPS7,** **PtTPS9,** **PtTPS10,** **PtTPS11,** **PtTPS12,** **PtTPS13 and PtTPS15.** The enzymes were expressed in *E. coli*, extracted, partially purified, and incubated with the substrates FPP, GPP and GGPP. Products were collected with a solid-phase microextraction (SPME) fiber and analyzed by GC-MS. 1, elemol; 2, β-eudesmol*; 3, unidentified sesquiterpene alcohol; 4, (*E*)-β-caryophyllene*; 5, α-humulene*; 6, β-elemene*; 7, eremophilene; 8, α-selinene*; 9, unidentified sesquiterpene; 10, γ-curcumene*; 11, nerolidol*; 12, (*E*)-β-ocimene*; 13, myrcene*; 14, limonene*; 15, terpinolene*; 16, linalool*; 17, sabinene*; 18, 1,8-cineole*; 19, terpineol*; 20, geranyllinalool*; cont., contamination. Compounds marked with * were identified using authentic standards.

All putative MTS (PtTPS6, PtTPS12, PtTPS13 and PtTPS15) accepted GPP as substrate and produced monoterpenes (Figure 2B). PtTPS6 formed (*E*)-β-ocimene as the major product with minor amounts of (*Z*)-β-ocimene. A similar narrow product specificity could also be observed for PtTPS15, which produced only linalool, and PtTPS12 which formed linalool and trace amounts of β-phellandrene, (*E*)-β-ocimene and α-terpinolene. However, PtTPS13 made five monoterpenes including α-pinene, β-pinene, sabinene, 1,8-cineole and α-terpineol. The incubation of PtTPS6 and PtTPS13 with FPP revealed no product formation. In contrast, PtTPS12 showed a broad sesquiterpene product spectrum and produced at least 25 different sesquiterpenes with γ-curcumene being the major one, and PtTPS15 was able to convert FPP to nerolidol (Figure 2A).

Four out of the 6 putative STS were able to convert FPP into different sesquiterpenes (Figure 2A). PtTPS9 produced (*E*)-β-caryophyllene and smaller amounts of α-humulene. In contrast, PtTPS11 showed a broader product spectrum comprising 18 different sesquiterpenes with β-elemene, eremophilene, α-selinene and an unidentified sesquiterpene representing the major peaks. Such complex product spectra could also be observed for PtTPS5, producing at least 27 different sesquiterpenes dominated by an unidentified sesquiterpene alcohol, and for PtTPS7 producing more than 15 different sesquiterpenes with elemol being the main product. Because the sesquiterpenes β-elemene (PtTPS11) and elemol (PtTPS7) are known to arise as thermal rearrangement products from germacrene A [25] and hedycaryol [26], respectively, during hot GC injection, the products of PtTPS7 and PtTPS11 were also analyzed using a colder GC injector (temperature, 150°C). Although β-elemene and elemol were still present in the GC chromatograms, an expansion of the germacrene A peak (PtTPS11) and hedycaryol peak (PtTPS7) could be observed, demonstrating the genuine activity of these enzymes (Additional file 1: Figure S6). In contrast to PtTPS5, PtTPS7, PtTPS9 and PtTPS11, the putative STS PtTPS14 showed only marginal activity with FPP and produced trace amounts of germacrene D (data not shown). PtTPS8, however, produced no sesquiterpenes. When offered GPP as a substrate, PtTPS11 formed several monoterpenes including myrcene, limonene, terpinolene and linalool (Figure 2B). Concerning the other STS, either trace activity (PtTPS5, PtTPS7, and PtTPS9) or no activity (PtTPS8, PtTPS14) was observed with GPP (data not shown). As predicted, PtTPS10 represented a DTS and was able to convert GGPP into geranyllinalool. Neither GPP nor FPP was accepted by PtTPS10. None of the other TPSs was able to accept GGPP as substrate.

A chiral analysis demonstrated that PtTPS9 formed exclusively the (−)-enantiomer of (*E*)-β-caryophyllene (Additional file 1: Figure S7A). For PtTPS15, the sesquiterpene product was (*3S*)-nerolidol while the monoterpene product was (*3S*)-linalool (Additional file 1: Figure S7B, S7C). A racemic mixture of (*3S*,*3R*)-linalool was made by PtTPS12 (Additional file 1: Figure S7D). PtTPS11 formed (−)-β-elemene (Additional file 1: Figure S7E) which corresponds to (+)-germacrene A as the actual enzyme product because the stereochemical configuration is retained at C7 [25].

### *TPS* gene expression is affected by herbivory

**Figure 3:**
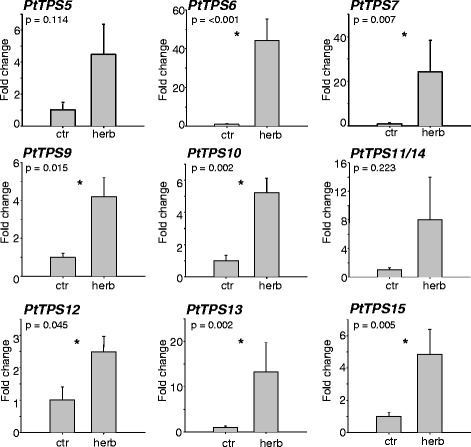
**Transcript abundance of terpene synthase genes in herbivore** **-damaged** **(herb)** **and undamaged control** **(ctr)** **leaves of** ***P. trichocarpa*** **.** Caterpillars were allowed to feed for 24 h on apical LPI3 (leaf plastochron index 3) leaves. Gene expression was determined by qRT-PCR. Means and standard errors are shown (n = 5). The student’s t-test was used to test for statistical significance. Asterisks indicate a significant difference between herbivore-infested and untreated control leaves. ctr, control treatment; herb, herbivory.

To analyze whether the expression of *PtTPS5*-*15* is influenced by herbivory, the transcript abundance of these genes was measured using qRT-PCR in apical, herbivore-damaged leaves (LPI3, for a detailed description of leaf plastochron index (LPI) see material and methods) compared to the respective undamaged leaves from control trees. The expression levels of *PtTPS5*-*15* generally increased after herbivore attack (Figure 3). Six of these genes were slightly upregulated, about 2- to 8-fold, with the increases in transcript accumulation significant for *PtTPS9*, *PtTPS10*, *PtTPS12* and *PtTPS15* but not for *PtTPS5* and *PtTPS11*/*14* (Figure 3, Additional file 2: Table S2). Repeated sequencing of amplicons from *PtTPS11*/*14*-qRT-PCR reactions revealed a 1:4 ratio of *PtTPS11* to *PtTPS14* transcript. A larger significant induction could be shown for *PtTPS*7 and *PtTPS13*, with 24.1-fold and 13.3-fold higher transcript abundance, respectively, in the damaged leaf compared to the undamaged control leaf (Figure 3). *PtTPS6* showed the strongest response to herbivore damage with a 44.1-fold increase in transcript abundance (Figure 3, Additional file 2: Table S2). No qRT-PCR analysis was performed for *PtTPS8* as no activity could be observed for the corresponding protein. Altogether the qRT-analysis showed distinct differences in the Δ*Cq* values (*Cq*_*TPS*_ – *Cq*_house-keeping gene_) for the identified *PtTPS* genes (Additional file 2: Table S3). *PtTPS11*/*14* and *PtTPS12* had Δ*Cq*-values higher than 15 indicating low expression levels. In contrast, small Δ*Cq*-values were observed for *PtTPS6* and *PtTPS9* indicating higher transcript abundance compared to the other *PtTPS* genes.

### Leaf age and position influence the quantitative composition of the herbivore-induced volatile blend

**Figure 4:**
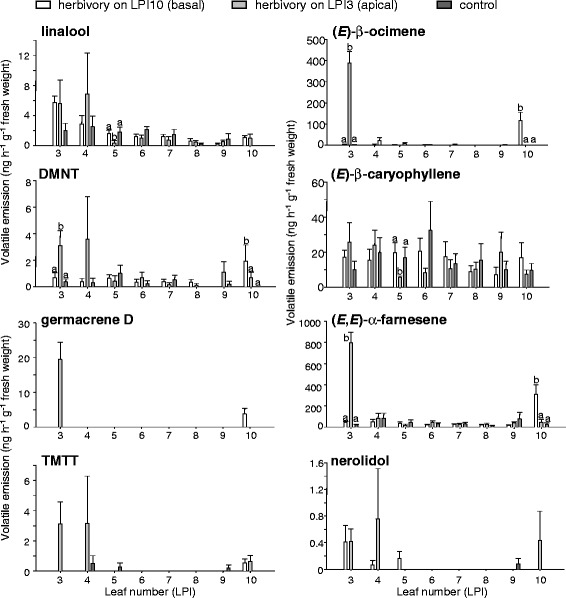
**Terpene emission from individual** ***P. trichocarpa*** **leaves after herbivory restricted to an apical or basal leaf or no herbivory.** Volatiles of eight single leaves, leaf plastochron indices 3 to 10 (LPI3 to LPI10) were measured from either control trees (ctr), trees which received herbivory apically on leaf LPI3 (ha, herbivory apical) or basally on leaf LPI10 (hb, herbivory basal). After separation by GC, the peaks were identified by MS and quantified using an FID. Means and standard errors are shown (n = 5). To test for statistical significance between the treatments in one leaf position, a one way analysis of variance (ANOVA) was performed. Different letters show significant differences. Germacrene D, TMTT and nerolidol were not tested for significance. L ratios and p values are given in Additional file 2: Table S6.

Our previous studies [10,21] already documented the complex volatile bouquet released from entire *P. trichocarpa* trees after damage by gypsy moth. To address the question of whether terpene emission is influenced by leaf age and position, we carried out a volatile collection from individual leaves (Additional file 1: Figure S8). A WAX (polyethyleneglycol) column with a length of 60 m was used for GC-MS analysis to ensure a better separation of the complex volatile mixtures. Single herbivore-damaged leaves emitted up to 78 different volatiles of which 58 could be identified (Additional file 2: Table S4). As previously described, (*E*)-β-ocimene, a monoterpene, and (*E*,*E*)-α-farnesene, a sesquiterpene likely produced by the sesquiterpene synthase PtTPS2, were the most abundant volatiles (Figure 4) [21]. Furthermore, the emission of (*Z*)-3-hexenol, 2-phenylethanol, benzaldehyde, benzyl cyanide and indole were also highly induced after herbivore feeding (Additional file 2: Table S4). In contrast to damaged leaves, neighboring non-infested leaves emitted only minor amounts of volatiles comparable to leaves from non-infested control trees, indicating that no systemic induction took place.

A comparison of the volatile blends released from uninfested leaves of different age stages (LPI3 to LPI10) revealed quantitative differences for single compounds within the tree (Additional file 2: Table S4). In undamaged control trees, linalool (p = 0.008, R^2^ = 0.18) and (*E*)-β-ocimene (p = 0.036, R^2^ = 0.12) were mainly emitted from younger apical leaves whereas (*Z*)-3-hexenol (p = 0.05, R^2^ = 0.1), nonanal (p = 0.007, R^2^ = 0.18) and nonanol (p = 0.09, R^2^ = 0.12) were more dominant in the volatile blends emitted from older basal leaves. However, (*E*)-β-caryophyllene (p = 0.87) and (*E*,*E*)-α-farnesene (p = 0.76) were emitted in similar amounts independent of leaf position (Figure 4, Additional file 2: Table S4). After herbivore infestation of a younger apical leaf (LPI3) or an older basal leaf (LPI10), this developmental difference became even clearer. Younger damaged leaves near the apex generally emitted more terpenes (apical 1297 ± 143 ng h^−1^ g^−1^ fresh weight vs. basal 475 ± 96; p = 0.001; t = −4.768; Figure 4, Additional file 2: Table S5), benzaldehyde (p = 0.008, T = 15) and the ester phenylethyl acetate (p = 0.003, t = −4.165) compared to older damaged leaves near the base (Additional file 2: Table S4). However, the alcohol nonanol was found to be released in higher quantities from older damaged leaves (p = 0.041, t = 2.437). Nitrogen-containing compounds displayed a similar induction pattern independent of the leaf age, except for phenylnitroethane (p = 0.008, T = 15) and indole (p = 0.004, t = −3.927), which were more abundant in younger damaged leaves, (Additional file 2: Table S4).

### The local and systemic emission of (*E*)-β-ocimene and (*E*)-β-caryophyllene can be correlated to the expression patterns of *PtTPS6* and *PtTPS9*, respectively

**Figure 5:**
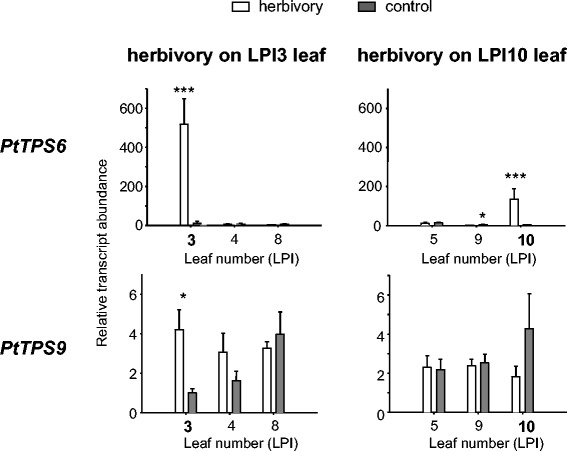
**Transcript abundance of** ***PtTPS6*** **and** ***PtTPS9*** **genes in individual poplar leaves.** Transcript abundance of *TPS* genes were measured in either control trees (ctr), trees which received herbivory apically on leaf LPI3 (ha, herbivory apical) or basally on leaf LPI10 (hb, herbivory basal). QRT-PCR was performed for herbivore-treated leaves (LPI3 and LPI10), their closest neighboring leaves (LPI4 and LPI9) and vascularly connected leaves (LPI8 and LPI5). Means and standard errors are shown (n = 5). The student’s t-test was used to test for statistical significance between the two treatments in one leaf. Asterisks indicate significant differences (p < 0.001, ** p < 0.01, * p < 0.05).

To determine whether local and systemic terpene emission pattern are reflected by *TPS* gene expression, the transcript abundance of the (*E*)-β-ocimene synthase PtTPS6 and the (*E*)-β-caryophyllene synthase PtTPS9 were measured in gypsy moth-damaged leaves (LPI3 and LPI10), the closest neighboring leaves (LPI4 and LPI9, respectively) and the leaves to which they are most closely vascularly connected (LPI8 and LPI5, respectively) [27,28]. *PtTPS6* gene expression was significantly increased in both herbivore-damaged LPI3 and LPI10 leaves. However, the induction was greater in the younger apical LPI3 leaf than in the older basal LPI10 leaf (Figure 5, Additional file 2: Table S2, S6). In contrast, *PtTPS9* was slightly induced in the apical LPI3 leaf and not induced in the basal LPI10 leaf (Figure 5, Additional file 2: Table S2, S6). Altogether, transcript abundance of *PtTPS*6 was strongly upregulated in herbivore-damaged leaves in comparison to the most adjacent leaves and vascularly connected leaves of the damaged tree as well as in comparison to all leaves measured on undamaged control trees. On the other hand, *PtTPS9* gene expression was hardly influenced by herbivory. No significant upregulation of *PtTPS9* transcripts in neighboring leaves or vascularly connected leaves could be detected (Figure 5, Additional file 2: Table S2, S6).

### The concentrations of jasmonates increased in herbivore-damaged leaves

**Figure 6:**
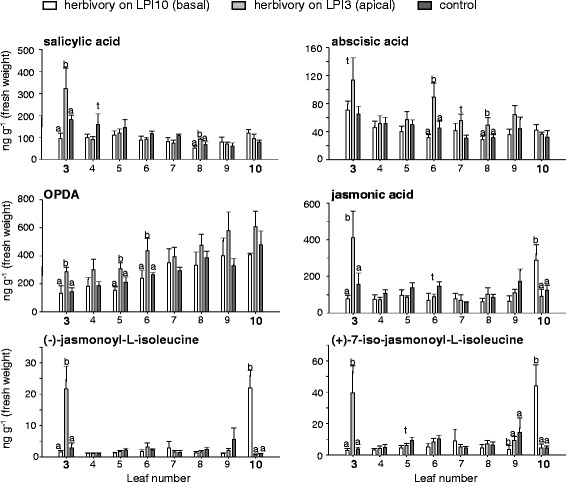
**Phytohormone concentrations in individual** ***P. trichocarpa*** **leaves after herbivory restricted to an apical or basal leaf or no herbivory.** Single leaves were measured from either control trees (ctr), trees which received herbivory apically on leaf LPI3 (ha, herbivory apical) or basally on leaf LPI10 (hb, herbivory basal). Means and standard errors are shown (n = 5). To test for statistical significance between the treatments in one leaf position, a one way analysis of variance (ANOVA) was performed. Different letters show significant differences. t indicates a trend (0.05 < p < 0.1).

As herbivore-induced volatile emission is commonly mediated by jasmonates and other phytohormones [29-31], we measured the concentrations of these compounds in individual leaves (LPI 3–10) of trees which suffered herbivory either on an apical leaf (LPI 3) or a basal leaf (LPI10). As illustrated in Figure 6, undamaged control trees showed a gradient for abscisic acid (ABA; R^2^ = 0.23, p = 0.001) and salicylic acid (SA; R^2^ = 0.4, p = 0.009) within the tree with higher concentrations in younger leaves. Conversely, 12-oxo-phytodienoic acid (OPDA) concentrations increased in older basal leaves (R^2^ = 0.55, p = <0.0001) while jasmonic acid (JA) and its isoleucine conjugates (JA-Ile), (−)-jasmonoyl-L-isoleucine and (+)-7-iso-jasmonoyl-L-isoleucine, were equally distributed throughout the tree (p = 0.44; p = 0.76; p = 0.94, respectively). Upon herbivore damage, the concentrations of JA and JA-Ile conjugates increased significantly and specifically in the damaged leaves independent of leaf position compared to undamaged leaves of control trees and leaves within the same tree (Figure 6, Additional file 2: Table S6). A significant induction of SA, ABA and OPDA occurred only after apical damage in damaged leaves or below (Figure 6, Additional file 2: Table S6).

## Discussion

### The *TPS*-*a* and *TPS*-*b* subfamilies in poplar are large and consist of members arisen by duplication events

Land plants generally possess a mid-size *TPS* gene family resulting from repeated gene duplication [12]. In this study we documented that the genome of *P. trichocarpa* contains 38 full length *TPS* genes in its current annotation (Figure 1). Hence, the poplar *TPS* gene family is comparable in size with the *TPS* gene families in *Arabidopsis* (32 *TPS* genes) [32], rice (*Oryza sativa*, 34 *TPS* genes) [33], sorghum (*Sorghum bicolor*, 24 *TPS* genes) [34] and tomato (29 *TPS* genes) [35]. In contrast, the genomes of grape (*Vitis vinifera*), white spruce (*Picea glauca*) and *S. moellendorfii* contain larger *TPS* gene families with 69, 69 and 66 putative functional *TPS* genes, respectively [12]. The *TPS* gene family in apple, however, is made up of only 10 putative functional *TPS* genes [36]. The majority of the poplar *TPSs* was found to cluster within the *TPS*-*a* and *TPS*-*b* subfamilies (Additional file 1: Figure S1). These clades generally contain *TPS* involved in sesquiterpene formation or in monoterpene formation, respectively [13]. In Arabidopsis multiple gene duplication events occurred within the *TPS*-*a*/*b* gene subfamilies [32]. The existence of multiple copies of *TPS*-*a*/*b* genes in poplar suggests a similar mechanism of gene duplication in this species. The high number of *TPS*-*a*/*b* genes stands in contrast to the two genes found in each of the poplar *TPS*-*c* and *TPS*-*e* subfamilies. These subfamilies commonly contain copalyl diphosphate synthases (CDS) and kaurene/kaurene-like synthases (KS/KSL), respectively, which can be involved in plant secondary as well as primary metabolism. Smaller TPS-c and TPS-e subfamilies were also described for conifer [37] and grape [38] and Arabidopsis contains only a single CDS and a single KS gene [32].

### PtTPS6, 9, 10, 12 and 15 may contribute to herbivore-induced volatile formation

The monoterpene synthase PtTPS6 was shown to produce (*E*)-β-ocimene, one of the most abundant compounds in the herbivore-induced volatile blend of poplar. Upon herbivory, a significant increase in *PtTPS6* transcript accumulation could be observed, suggesting a role of PtTPS6 in herbivore-induced (*E*)-β-ocimene formation *in planta* (Figures 4, 5). (*E*)-β-ocimene can be produced by a large number of plant species in floral scent [39], as a herbivore-induced volatile of leaves, e.g. tobacco (*Nicotiana spp*), corn (*Zea mays*), cotton (*Gossypium hirsutum*) and Arabidopsis plants [5,9,40,41], and also as part of essential oils, e.g. chamomile (*Matricaria recutita*) [42]. When added to the spider mite-induced volatile blend of wishbone flower (*Torenia hybrida*), (*E*)-β-ocimene was able to enhance the blend’s ability to attract predatory mites [43]. In contrast, a repellent effect of (*E*)-β-ocimene was observed for another herbivore enemy, *Glyptapanteles liparidis*, a parasitoid of the gypsy moth larvae [7]. Besides its effect on insects, (*E*)-β-ocimene can also function in plant-plant communication by mediating the induction of defense related genes [44,45]. Thus the (*E*)-β-ocimene synthase PtTPS6 might play different roles in direct or indirect defense of poplar.

Linalool, another prominent constituent of the poplar volatile bouquet, can be produced by PtTPS3 and PtTPS12 [21] (Figure 2). While PtTPS3 formed exclusively (+)-(*3S*)-linalool, PtTPS12 produced a racemic linalool mixture (Additional file 1: Figure S7). As poplar emits (+)-(*3S*)-linalool as well as (−)-(*3R*)-linalool [21], both enzymes may contribute to linalool formation in the tree. The upregulation of *PtTPS12* transcripts additionally supports the function of PtTPS12 in herbivore-induced volatile biosynthesis (Figure 3). Interestingly, PtTPS12 possessed also sesquiterpene synthase activity *in vitro* and formed a complex mixture of sesquiterpenes (Figure 2). However, a transit peptide at the N-terminus of PtTPS12 suggests a plastidal localization and thus an *in vivo* function as monoterpene synthase in accordance with the absence of PtTPS12 sesquiterpene products in the poplar volatile blend.

*PtTPS15* was shown to encode a nerolidol synthase (Figure 2). This TPS clusters within the TPS-g family together with PtTPS3 (Additional file 1: Figure S4) [21]. Both terpene synthases are characterized by the absence of the RR(x)_8_W-motif, which is a typical feature of members of the TPS-g clade [35]. PtTPS15 and PtTPS3 produce (+)-(*3S*)-nerolidol *in vitro*, the enantiomer also present in the poplar scent (Additional file 1: Figure S7) [21]. In contrast to PtTPS3, PtTPS15 does not have a transit peptide and is therefore more likely responsible for (+)-(*3S*)-nerolidol biosynthesis *in planta*. Nerolidol has been shown to be converted into the homoterpene DMNT which is considered to play an important role in plant defense [12]. Danner and coworkers (2011) already speculated about the involvement of the (−)-(*3R*)-nerolidol-producing PtTPS4 in herbivore-induced DMNT biosynthesis. As the expression of *PtTPS4* was only slightly induced after herbivore attack, the existence of another nerolidol synthase was predicted. Since transcript accumulation of *PtTPS15* was strongly upregulated after herbivore damage, it is likely that PtTPS15 contributes to the production of the precursor for DMNT biosynthesis *in planta* (Figure 3). TMTT is another homoterpene emitted by *P. trichocarpa*. The precursor of TMTT, geranyllinalool, was produced by the diterpene synthase PtTPS10 (Figure 2). The transcript level of *PtTPS10* increased after herbivore damage which corresponded to the induced emission of TMTT (Figure 3). Both DMNT and TMTT are described to be emitted from several plants including maize, lima bean, Arabidopsis and tomato [46,47] and a role of TMTT and DMNT in, for example, predatory mite attraction in tomato and Arabidopsis, respectively, has been demonstrated [48,49].

The volatile (*E*)-β-caryophyllene has been reported to attract enemies of both above- and below-ground maize herbivores [50]. In our study, (*E*)-β-caryophyllene emission was not significantly increased after herbivore damage but showed a trend to higher emission from damaged leaves corresponding to a small increase in (*E*)-β-caryophyllene synthase (*PtTPS9*) transcripts (Figures 4, 5). However, Danner and coworkers (2011) described (*E*)-β-caryophyllene as an herbivore-inducible poplar volatile. Plant age, greenhouse conditions and previous infections could have led to already increased levels of (*E*)-β-caryophyllene in plants used for the present study.

1,8-Cineole, the main product of PtTPS13, was only present in trace amounts in the herbivore-induced volatile blend of *P. trichocarpa* (Figure 2, Additional file 2: Table S4). A low transcript abundance (indicated by a high Δ*Cq*-value, Additional file 2: Table S3) is likely to be the reason for this low emission (Figure 3). The *in vitro* products of PtTPS5, PtTPS7 and PtTPS11 (sesquiterpene alcohols and germacrene A, Figure 2) could not be detected in the herbivore-induced poplar volatile blend, although qRT-PCR analysis revealed a transcriptional upregulation in herbivore-damaged leaves in comparison to undamaged control leaves (Figure 3). Low transcript abundance (as indicated for *PtTPS11* by a high Δ*Cq*-value; Additional file 2: Table S3), but also high *K*_*m*_ values for FPP or low turnover numbers or lifetimes for the respective proteins could explain the lack of the respective *in vitro* products of the enzymes in the poplar volatile blend. Or, the products could be further converted into non-volatile metabolites, such as sesquiterpene phytoalexins. There are reports of sesquiterpene synthases involved in phytoalexin biosynthesis [51-55]. In maize, for example, the existence of an herbivore-inducible β-macrocarpene synthase and the absence of this sesquiterpene in the volatile blend led to the discovery of β-macrocarpene-derived phytoalexins [50,55]. Germacrene A, the major *in vitro* product of PtTPS11, was shown to be the precursor for sesquiterpene lactones in various plants like chicory (*Cichorium intybus*) and pyrethrum (*Tanacetum cinerariifolium*) [56-58]. Hence, we propose that sesquiterpene alcohols produced by PtTPS5 and PtTPS7 and germacrene A produced by PtTPS11 are further metabolized in poplar.

Altogether, out of the 38 putative TPS encoded in the poplar genome, 11 TPS enzymes could be functionally characterized in this study. Including the 4 previously published mono- and sesquiterpene synthases, the germacrene D synthase PtTPS1, the (*E*,*E*)-α-farnesene synthase PtTPS2, and the linalool/nerolidol synthases PtTPS3 and PtTPS4 [21] as well as the isoprene synthase [22], about half of the *TPS* genes present in the genome have now been characterized and their enzymatic function *in vitro* determined. Nearly all of the terpenes emitted by poplar after herbivory can be explained by the enzyme activities of the 15 so far characterized mono-, sesqui- and diterpene synthases (Additional file 2: Table S8). Nevertheless it is possible that additional *TPS* genes are expressed in other organs of *P. trichocarpa* as it was already described for other plants. For example, *TPS* genes specifically expressed in flowers or roots were described for chamomile and Arabidopsis [42,59,60].

### Herbivore-induced terpene emission in *P. trichocarpa* is restricted to the damaged leaf and likely mediated by jasmonates

A comparison of the qualitative and quantitative composition of the volatile blends released locally from herbivore-damaged leaves and systemically from undamaged adjacent leaves has been described for *P. nigra* and hybrid poplar (*P. trichocarpa* x *deltoides*) [7,20]. In both studies, a systemic volatile induction in the undamaged leaves above the site of herbivore-damage was reported. On the contrary, we showed that no systemic induction of volatiles was occurring at the level of an individual leaf in *P. trichocarpa* (Additional file 2: Table S4, S5). As already observed in maize [61], volatile emission was restricted to the damaged leaf. This specific local volatile emission could be important in intra-plant signaling and provides a reliable short-range signal for parasitoids searching for their hosts.

Jasmonic acid and their amino acid conjugates are well known as mediators of herbivore-induced plant defenses, including the emission of volatiles in many plant species [31,62]. In a recent publication, Clavijo McCormick and coworkers (2014) demonstrated that jasmonates were upregulated after herbivory in black poplar. Moreover, exogenous application of JA to *P. nigra* and *P. trichocarpa* leaves resulted in increased volatile emission [7] (Additional file 2: Table S9) and a transcriptome analysis of *P. trichocarpa* x *deltoides* leaves treated with forest tent caterpillars (*Malocosoma disstria*) revealed an upregulation of genes involved in both jasmonate biosynthesis as well as volatile formation [63]. The herbivore-induced local terpene emission of *P. trichocarpa* was also reflected by increased levels of *TPS* transcripts and jasmonates in the damaged leaves in comparison to undamaged adjacent leaves (Figures 5, 6). Therefore JA and JA-Ile conjugates appear to be major signals for transcriptional regulation of volatile biosynthesis in *P. trichocarpa*.

Although volatile emission of black poplar is inducible by JA application but not by SA application, SA levels are increased after herbivore feeding [7]. In our experiments with *P. trichocarpa*, only herbivory on younger apical leaves resulted in significantly increased SA concentrations in damaged leaves (Figure 6). Interestingly, elevated levels of SA were also detected in the vascular-connected leaf (LPI of the herbivore-damaged leaf + 5) [27,28] beneath the herbivore-damaged leaf (Figure 6). Additionally, apical herbivory resulted in increased concentrations of OPDA and ABA in basal leaves (Figure 6). A mobile signal travelling from the apical leaves downward could explain these observations and would provide a reasonable mechanism for the basipetal, systemic defense reactions already described for poplar [64].

## Conclusions

In this study we investigated the terpene synthase gene family in *P. trichocarpa* and its contribution to herbivore-induced volatile formation. The production of volatile terpenes in poplar is mainly regulated by transcript accumulation of multiple *TPS* genes. The restriction of volatile release to the damaged leaf might play a role in intra-plant signaling and help herbivore enemies to better find their hosts or preys in the tree canopy. While about one third of all poplar TPSs seem to be involved in herbivore-induced volatile terpene production, the function of other TPS is still unclear. They might play roles in other defense reactions in the different organs of poplar trees.

## Methods

### Plant and insect material

Western balsam poplar (*Populus trichocarpa*) trees were propagated from monoclonal stem cuttings (clone 625, NW-FVA, Hann. Münden, Germany) and grown under summer conditions in the greenhouse (24°C, 60% rel. humidity, 16 h/8 h light/dark cycle) in a 1:1 mixture of sand and soil (Klasmann potting substrate), until they reached about 1 m in height. Gypsy moth (*Lymantria dispar*) egg batches were kindly provided by Hannah Nadel, APHIS, USA. After hatching, the caterpillars were reared on an artificial diet (Gypsy moth diet, MP Biomedicals LLC, Illkirch, France).

### Poplar volatile collection and analysis

To investigate the spatial distribution of volatile emission after gypsy moth feeding, we conducted single-leaf volatile collections. Eight leaves (from LPI3 to LPI10, LPI = leaf plastochron index [28]) from each tree were individually enclosed with a PET bag (“Bratschlauch”, Toppits, Minden, Germany) by fixing the ends with cable binders. Trees were infested with *L. dispar* larvae on either an apical leaf (LPI3) or a basal leaf (LPI10) and volatiles were separately collected from leaves LPI3 to LPI10 (Additional file 1: Figure S8). Five *L. dispar* caterpillars in third to fourth instar starved for 12 h were released on the leaves. The caterpillars were fed with poplar leaves for one week *prior* to the onset of the experiment. Caterpillars were allowed to feed for 24 h (16.00 (day 1) – 16.00 h (day 2)). Volatiles were collected for 6 hours during the middle of the light period of day 2 (10.00 - 16.00 h) after caterpillar removal using a push-pull system as described by Tholl et al. (2006) [65]. Air flow was maintained in the system through teflon tubes. Charcoal-filtered air was constantly pumped into the bags at a flow rate of 1.5 l/min. The outgoing air (flow: 1 l/min) passed a filter packed with 30 mg Super Q (ARS, Inc., Gainesville, FL, USA) to adsorb the volatile compounds. After collection the volatiles were desorbed by eluting the filter twice with 100 μL dichloromethane containing nonyl acetate as an internal standard (10 ng μL^−1^). Five replicates each of LPI3-treated trees, LPI10-treated trees and undamaged control trees were measured.

Qualitative and quantitative volatile analysis was conducted using an Agilent 6890 Series gas chromatograph coupled to an Agilent 5973 quadrupole mass selective detector (interface temp, 250°C; quadrupole temp, 150°C; source temp, 230°C; electron energy, 70 eV) or a flame ionization detector (FID) operated at 300°C, respectively. The constituents of the volatile bouquet were separated using a ZB-WAX column (Phenomenex, Aschaffenburg, Germany, 60 m × 0.25 mm × 0.15 μm) and He (MS) or H_2_ (FID) as carrier gas. The sample (1 μL) was injected without split at an initial oven temperature of 40°C. The temperature was held for 2 min and then increased to 225°C with a gradient of 5°C min^−1^, held for another 2 min and then further increased to 250°C with a gradient of 100°C min^−1^ and a hold of 1 min. Compounds were identified by comparison of retention times and mass spectra to those of authentic standards obtained from Fluka (Seelze, Germany), Roth (Karlsruhe, Germany), Sigma (St. Louis, MO, USA), Bedoukian (Danbury, CT, USA), or by reference spectra in the Wiley and National Institute of Standards and Technology libraries. Aldoxime standards were synthesized as described in Irmisch et al. 2013 [10].

### Plant tissue sampling, RNA extraction and reverse transcription

Poplar material was harvested after 24 h of herbivory, flash-frozen with liquid nitrogen and stored at −80°C until further processing. After grinding, the total RNA was isolated using an Invisorb Spin Plant RNA Mini Kit (Invitek GmbH, Berlin, Germany) according to the manufacturer’s instructions. RNA concentration, purity and quality were accessed using a spectrophotometer (NanoDrop 2000c, Thermo Scientific, Wilmington, USA) and an Agilent 2100 Bioanalyzer (Agilent Technologies GmbH, Waldbronn, Germany). Prior to cDNA synthesis, 0.75 μg RNA was DNase-treated using 1 μL DNase (Fermentas GmbH, St. Leon Roth, Germany). Single-stranded cDNA was prepared from the DNase-treated RNA using SuperScript™ III reverse transcriptase and oligo (dT_12–18_) primers (Invitrogen, Carlsbad, CA, USA).

### Identification and isolation of *PtTPS* genes

To identify putative poplar *TPS* genes, a BLAST search against the *P. trichocarpa* genome database (http://www.phytozome.net/poplar) using PtTPS1 (JF449450) and AtGAS2 (Q9SAK2) as inqury sequences was conducted. Thirty-eight full length *TPS* genes, having a minimum length of 520 amino acids were identified. Fifteen (including *PtTPS1*-*4*) [21] of these sequences could be amplified from cDNA obtained from herbivore-damaged leaves of *P. trichocarpa*. Primer sequence information is available in Additional file 2: Table S10. PCR products were cloned into the sequencing vector pCR®^−^Blunt II-TOPO® (Invitrogen) and both strands were fully sequenced. Signal peptide prediction was done using the TargetP 1.1 server (http://www.cbs.dtu.dk/services/TargetP/) and a positive prediction was taken if the reliability class (RC-value) was greater than 4. Sequences were deposited in GenBank with the accession numbers KF776503 (*PtTPS5*), KF776504 (*PtTPS6*), KF776505 (*PtTPS7*), KF776506 (*PtTPS8*), KF776507 (*PtTPS9*), KF776508 (*PtTPS10*), KF776509 (*PtTPS11*), KF776510 (*PtTPS12*), KF776511 (*PtTPS13*), KF776512 (*PtTPS14*), KF776502 (*PtTPS15*).

### Heterologous expression of PtTPS in *E. coli*

The open reading frames of the *TPS* genes were cloned as *Bsa*I fragments into the pASK-IBA7 vector (IBA-GmbH, Göttingen, Germany). The *E. coli* strain TOP10 (Invitrogen) was used for expression. Cultures were grown at 37°C, induced at an OD_600_ = 0.6 with 200ug/l anhydrotetracycline (IBA-GmbH) and subsequently placed at 18°C and grown for another 20 hours. The cells were collected by centrifugation and disrupted by a 3 × 30 s treatment with a sonicator (Bandelin UW2070, Berlin, Germany) in chilled extraction buffer (50 mM Mopso, pH 7.0, with 5 mM MgCl_2_, 5 mM sodium ascorbate, 0.5 mM PMSF, 5 mM dithiothreitol and 10% (v/v) glycerol). Cell fragments were removed by centrifugation at 14,000 g and the supernatant was desalted into assay buffer (10 mM Mopso, pH 7.0, 1 mM dithiothreitol, 10% (v/v) glycerol) by passage through a Econopac 10DG column (BioRad, Hercules, CA, USA).

### Analysis of recombinant PtTPS

To determine the catalytic activity of the different terpene synthases, enzyme assays containing 40 μl of the bacterial extract and 60 μl assay buffer with 10 μM (*E*,*E*)-FPP or (*E*)-GPP and 10 mM MgCl_2_, in a Teflon-sealed, screw-capped 1 ml GC glass vial were performed. A SPME (solid phase microextraction) fiber consisting of 100 μm polydimethylsiloxane (SUPELCO, Belafonte, PA, USA) was placed into the headspace of the vial for 45 min incubation at 30°C. For analysis of the adsorbed reaction products, the SPME fiber was directly inserted into the injector of the gas chromatograph. To determine diterpene synthase activity, assays were set up as described above, containing 50 μM (*E*,*E*,*E*)-GGPP as substrate, and overlaid with 100 μl hexane. After incubation for 60 min at 30°C the hexane phase was collected and analyzed using GC-MS.

As a negative control, we incubated raw protein extracts from *E. coli* expressing the empty vector pASK-IBA7 with the substrates GPP, FPP, and GGPP, respectively, as described above. As shown in Additional file 1: Figure S9, no terpene hydrocarbon or terpene alcohols were formed with the exception of a very few GPP and FPP hydrolysis products which were probably produced by unspecific *E. coli* phosphatases.

The TPS enzyme products were analyzed and identified using GC-MS as described above for poplar volatiles. The GC was operated with a DB-5MS column (Agilent, Santa Clara, USA, 30 m × 0.25 mm × 0.25 μm). The sample (SPME) was injected without split at an initial oven temperature of 50°C. The temperature was held for 2 min, then increased to 240°C with a gradient of 7°C min^−1^, and further increased to 300°C with a gradient of 60°C min^−1^ and a hold of 2 min. For the GC-MS analysis with a cooler injector, the injector temperature was reduced from 220°C to 150°C. For volatile diterpene analysis, 2 μl of the hexane samples were injected without split at an initial oven temperature of 80°C. The temperature was held for 2 min, than increased to 250°C with a gradient of 7°C min^−1^, and further increased to 320°C with a gradient of 100°C min^−1^ and a hold of 2 min.

Chiral GC-MS analysis was performed using a Rt™-βDEXsm-column (Restek, Bad Homburg, Germany) and a temperature program from 50°C (2 min hold) at 2°C min^−1^ to 220°C (1 min hold). Enantiomers were identified using authentic standards obtained from (Fluka, Seelze, Germany). A racemic mixture of (*E*)-β-caryophyllene was kindly provided by Stefan Garms (MPI for Chemical Ecology, Jena, Germany). A (+)-germacrene A synthase from chamomile MrTPS3 (*Matricaria recutita*) [42] was used to prepare an authentic (+)-germacrene A standard.

### qRT-PCR analysis of *PtTPS* expression

RNA was isolated from leaves treated for 24 h with herbivory and cDNA was prepared as described above and diluted 1:3 with water. For the amplification of *TPS* gene fragments with a length between 110–200 bp, a gene specific primer pair was designed having a T_m_ of about 60°C, a GC content between 40 - 55% and a primer length in the range of 19–25 nt (Additional file 2: Table S9). Due to the high sequence similarity, only one primer pair was used for the amplification of *PtTPS11* and *PtTPS14*. Sequencing of PCR products obtained from four biological replicates revealed the average percentage for the transcripts of *PtTPS11* and *PtTPS14*.

Primer specificity was confirmed by agarose gel electrophoresis, melting curve analysis and standard curve analysis and by sequence verification of cloned PCR amplicons. *Ubiquitin* was used as a reference gene [66]. Samples were run in triplicates using Brilliant® III SYBR® Green QPCR Master Mix (Stratagene, CA, USA) with ROX as reference dye. The following PCR conditions were applied for all reactions: Initial incubation at 95°C for 3 min followed by 40 cycles of amplification (95°C for 20 sec, 60°C for 20 sec). Plate reads were taken during the annealing and the extension step of each cycle. Data for the melting curves were recorded at the end of cycling from 55°C to 95°C.

All samples were run on the same PCR machine (MxPro – Mx3000P, Stratagene, Agilent Technologies, USA) in an optical 96-well plate. Five biological replicates were analyzed as triplicates in the qRT-PCR for each of the three treatments. Data for the relative quantity to calibrator average (dRn) were exported from the MXPro Software.

### Phylogenetic tree reconstruction

For the estimation of a phylogenetic tree, we used the MUSCLE algorithm (gap open, −2.9; gap extend, 0; hydrophobicity multiplier, 1.5; clustering method, upgmb) implemented in MEGA5 [67] to compute an amino acid alignment of all full length poplar TPS enzymes. Based on the MUSCLE alignment, the tree was reconstructed with MEGA5 using a neighbor-joining algorithm (Poisson model). A bootstrap resampling analysis with 1000 replicates was performed to evaluate the tree topology. Following this procedure, a phylogenetic tree containing the characterized PtTPS enzymes and other representative TPS enzymes was constructed. An additional tree showing the phylogenetic relation of all full length *PtTPS* genes and representative *TPS* genes from other plants was constructed using a clustal W alignment (nucleotides treated as triplicates) to build up a neighbor-joining tree using MEGA 5 as described above.

An alignment of all characterized PtTPS enzymes and an alignment of the putative diterpene synthases of poplar with other characterized diterpene synthases were constructed and visualized using BioEdit (http://www.mbio.ncsu.edu/bioedit/bioedit.html) and the ClustalW algorithm.

### Quantification of Jasmonic acid, JA-Ile conjugates, OPDA, ABA and Salicylic acid

For phytohormone analysis, 110 mg of finely ground leaf material was extracted with 1 ml of methanol containing 60 ng of 9,10-D_2_-9,10-dihydrojasmonic acid , 60 ng D_4_-salicylic acid (Sigma-Aldrich), 60 ng D_6_-abscisic acid (Santa Cruz Biotechnology, Santa Cruz, CA, USA), and 15 ng of jasmonic acid-[^13^C_6_] isoleucine conjugate as internal standards. Jasmonic acid-[^13^C_6_] isoleucine conjugate was synthesized as described by Kramell et al. (1988) using [^13^C_6_] Isoleucine (Sigma-Aldrich) [68].

Chromatography was performed on an Agilent 1200 HPLC system (Agilent Technologies). Separation was achieved on a Zorbax Eclipse XDB-C18 column (50 × 4.6 mm, 1.8 μm, Agilent). Formic acid (0.05%) in water and acetonitrile were employed as mobile phases A and B, respectively. The elution profile was: 0–0.5 min, 5% B; 0.5-9.5 min, 5-42% B; 9.5-9.51 min 42-100% B; 9.51-12 min 100% B and 12.1-15 min 5% B. The mobile phase flow rate was 1.1 ml/min. The column temperature was maintained at 25°C. An API 3200 tandem mass spectrometer (Applied Biosystems) equipped with a Turbospray ion source was operated in negative ionization mode. The instrument parameters were optimized by infusion experiments with pure standards, where available. The ion spray voltage was maintained at 4500 eV. The turbo gas temperature was set at 700°C. Nebulizing gas was set at 60 psi, curtain gas at 25 psi, heating gas at 60 psi and collision gas at 7 psi. Multiple reaction monitoring (MRM) was used to monitor analyte parent ion → product ion: *m*/*z* 136.9 → 93.0 (collision energy (CE )-22 V; declustering potential (DP) -35 V) for salicylic acid; *m*/*z* 140.9 → 97.0 (CE −22 V; DP −35 V) for D_4_-salicylic acid; *m*/*z* 209.1 → 59.0 (CE −24 V; DP −35 V) for jasmonic acid; *m*/*z* 213.1 → 56.0 (CE −24 V; DP −35 V) for 9,10-D2-9,10-dihydrojasmonic acid; *m*/*z* 263.0 → 153.2 (CE −22 V; DP −35 V) for abscisic acid; *m*/*z* 269.0 → 159.2 (CE −22 V; DP −35 V) for D_6_-abscisic acid; *m*/*z* 322.2 → 130.1 (CE -30 V; DP -50 V) for jasmonic acid-isoleucine conjugate; *m*/*z* 328.2 → 136.1 (CE -30 V; DP -50 V) for jasmonic acid-[^13^C_6_]-isoleucine conjugate. Both Q1 and Q3 quadrupoles were maintained at unit resolution. Analyst 1.5 software (Applied Biosystems) was used for data acquisition and processing. Linearity in ionization efficiencies was verified by analyzing dilution series of standard mixtures. Phytohormones were quantified relative to the signal of their corresponding internal standard. For quantification of 12-oxophytodienoic acid, cis-OPDA, 9,10-D_2_-9,10-dihydrojasmonic acid was used as the internal standard applying an experimentally determined response factor of 2.24.

### Statistical analysis

Whenever necessary, the data were log transformed to meet statistical assumptions such as normality and homogeneity of variances. Throughout the manuscript data are presented as means ± SE. To compare *P. trichocarpa* volatile emission of herbivore-induced leaves and their respective control leaves, emission from apical vs. basal leaves, and expression of *TPS* in leaves from herbivore-induced trees vs. undamaged controls, Student’s t-tests were performed with SigmaPlot 11.0 for Windows (Systat Software Inc. 2008). A linear regression was performed using R2.15.2 (R Development Core Team, http://www.R-project.org) to check for gradients of volatile emission on the trees. To compare phytohormone concentrations in single leaves of *P. trichocarpa* and *PtTPS6* and *PtTPS9* gene expression in different single leaves of one tree, mixed-effects models (nlme package) with leaf number as fixed effect and plant identity as random effect were used, followed by a maximum likelihood ratio test. The testing for significant differences in phytohormone content and terpene emission between the apical, basal and control treatment in one leaf position was done using a one way analysis of variance (ANOVA). Models were simplified by factor level reduction [69], and were done with R2.15.2.

### Availability of supporting data

Phylogenetic data (trees and the data used to generate them) have been deposited in TreeBASE respository and are available under the URL http://purl.org/phylo/treebase/phylows/study/TB2:S16450.

## Additional files

Additional file 1:
**This file contains 9 supplemental figures.**
Additional file 2:
**This file contains 10 supplemental tables.**
