# Supplementary material for: Terpene synthases and their contribution to herbivore-induced volatile emission in western balsam poplar (Populus trichocarpa)
Source: BMC Plant Biol. 2014 Oct 11;14:270. doi: 10.1186/s12870-014-0270-y (PMC4197230; doi:10.1186/s12870-014-0270-y)
Supplement: Additional file 1: — This file contains 9 supplemental figures. [file 12870_2014_270_MOESM1_ESM.pptx]

## Slide 1
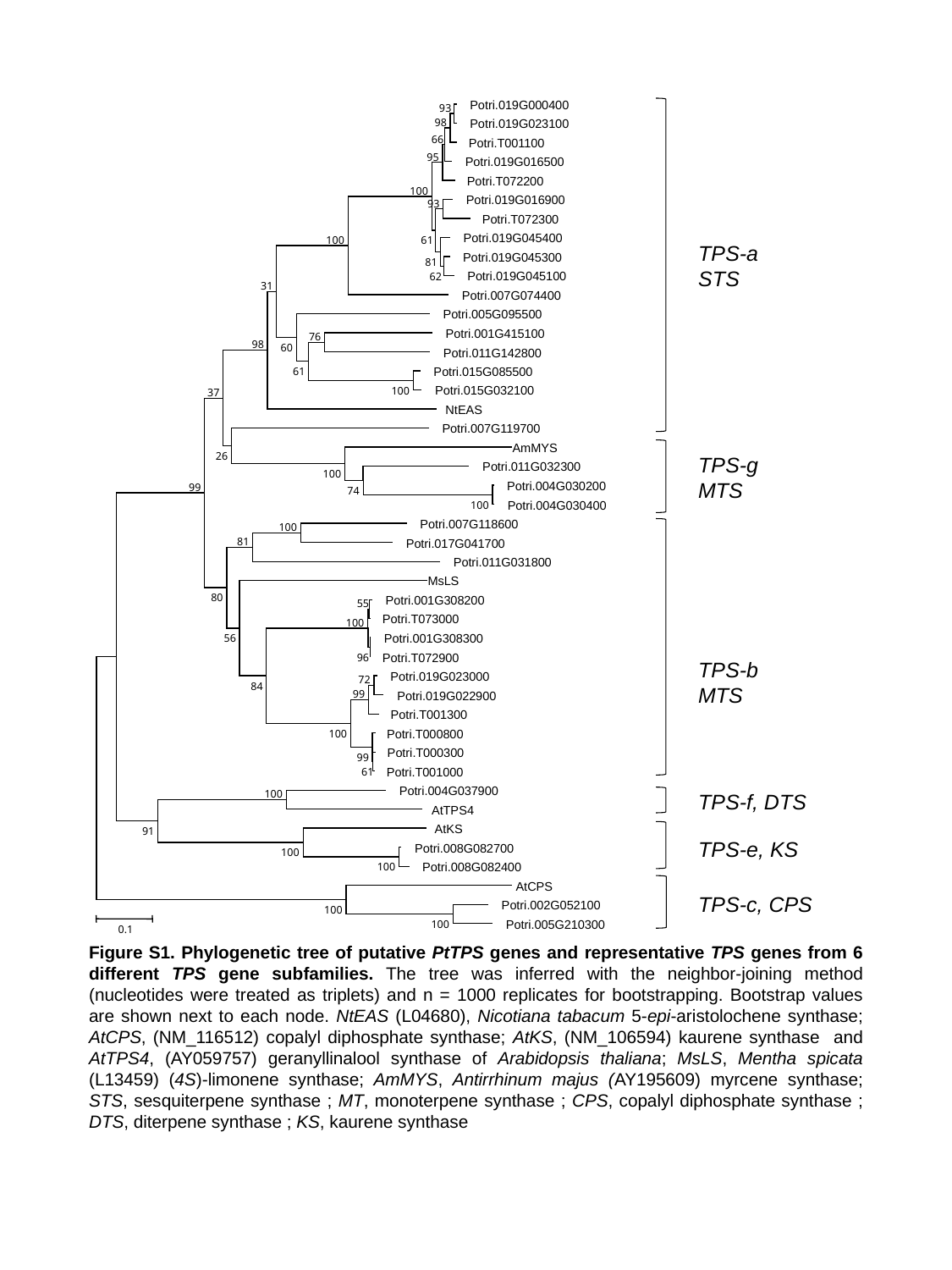

Potri.019G000400
93
98
 Potri.019G023100
66
 Potri.T001100
95
 Potri.019G016500
 Potri.T072200
100
 Potri.019G016900
93
 Potri.T072300
 Potri.019G045400
100
TPS-a
STS
61
 Potri.019G045300
81
 Potri.019G045100
62
31
 Potri.007G074400
 Potri.005G095500
 Potri.001G415100
76
98
60
 Potri.011G142800
 Potri.015G085500
61
 Potri.015G032100
100
37
 NtEAS
 Potri.007G119700
AmMYS
TPS-g
MTS
26
 Potri.011G032300
100
 Potri.004G030200
99
74
 Potri.004G030400
100
 Potri.007G118600
100
81
 Potri.017G041700
 Potri.011G031800
MsLS
80
 Potri.001G308200
55
 Potri.T073000
100
 Potri.001G308300
56
 Potri.T072900
TPS-b
MTS
96
 Potri.019G023000
72
84
99
 Potri.019G022900
 Potri.T001300
 Potri.T000800
100
 Potri.T000300
99
 Potri.T001000
61
TPS-f, DTS
 Potri.004G037900
100
 AtTPS4
 AtKS
91
TPS-e, KS
 Potri.008G082700
100
 Potri.008G082400
100
 AtCPS
TPS-c, CPS
 Potri.002G052100
100
 Potri.005G210300
100
0.1
Figure S1. Phylogenetic tree of putative PtTPS genes and representative TPS genes from 6 different TPS gene subfamilies. The tree was inferred with the neighbor-joining method (nucleotides were treated as triplets) and n = 1000 replicates for bootstrapping. Bootstrap values are shown next to each node. NtEAS (L04680), Nicotiana tabacum 5-epi-aristolochene synthase; AtCPS, (NM_116512) copalyl diphosphate synthase; AtKS, (NM_106594) kaurene synthase and AtTPS4, (AY059757) geranyllinalool synthase of Arabidopsis thaliana; MsLS, Mentha spicata (L13459) (4S)-limonene synthase; AmMYS, Antirrhinum majus (AY195609) myrcene synthase; STS, sesquiterpene synthase ; MT, monoterpene synthase ; CPS, copalyl diphosphate synthase ; DTS, diterpene synthase ; KS, kaurene synthase

## Slide 2
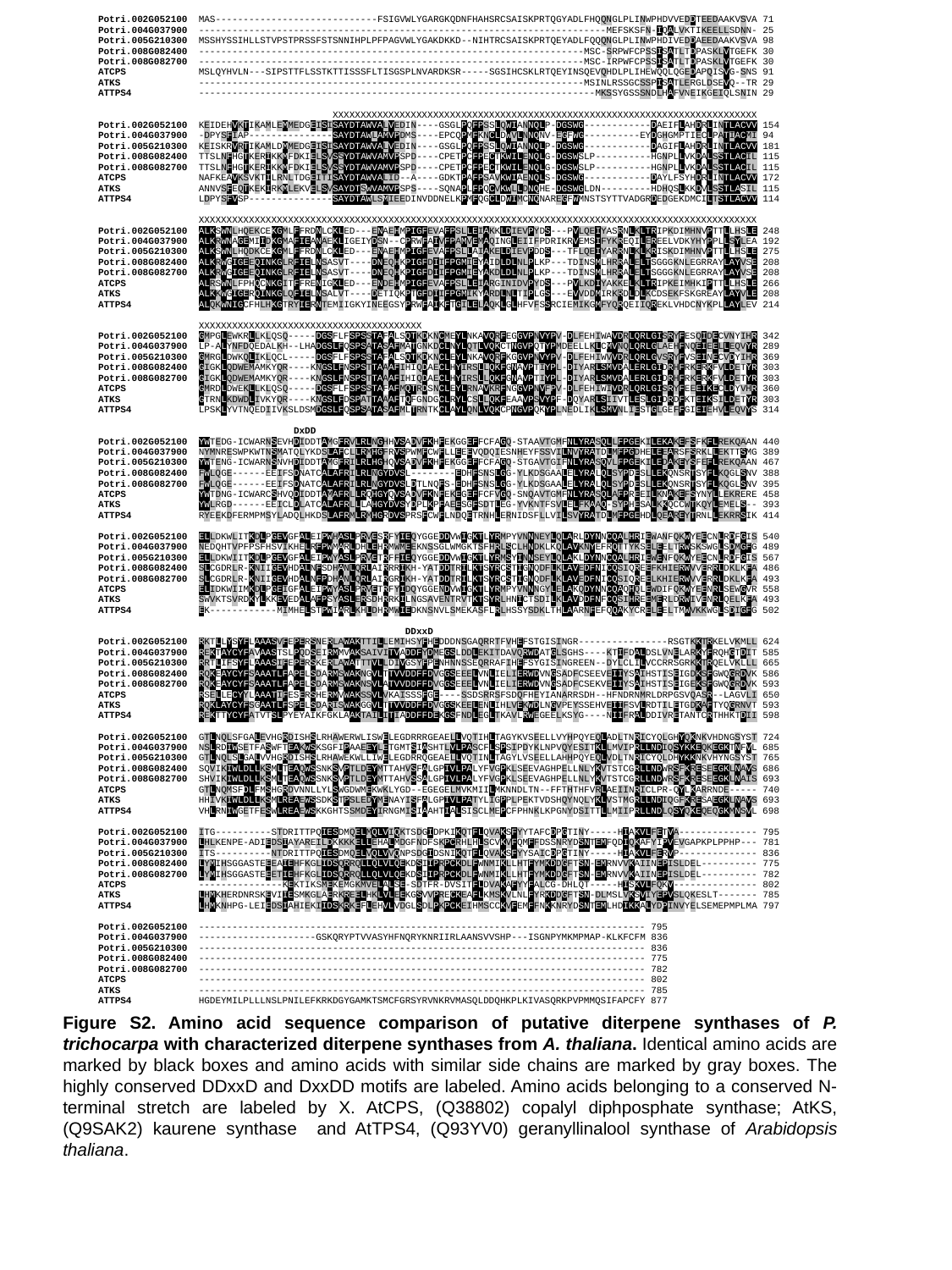

Figure S2. Amino acid sequence comparison of putative diterpene synthases of P. trichocarpa with characterized diterpene synthases from A. thaliana. Identical amino acids are marked by black boxes and amino acids with similar side chains are marked by gray boxes. The highly conserved DDxxD and DxxDD motifs are labeled. Amino acids belonging to a conserved N-terminal stretch are labeled by X. AtCPS, (Q38802) copalyl diphposphate synthase; AtKS, (Q9SAK2) kaurene synthase and AtTPS4, (Q93YV0) geranyllinalool synthase of Arabidopsis thaliana.

## Slide 3
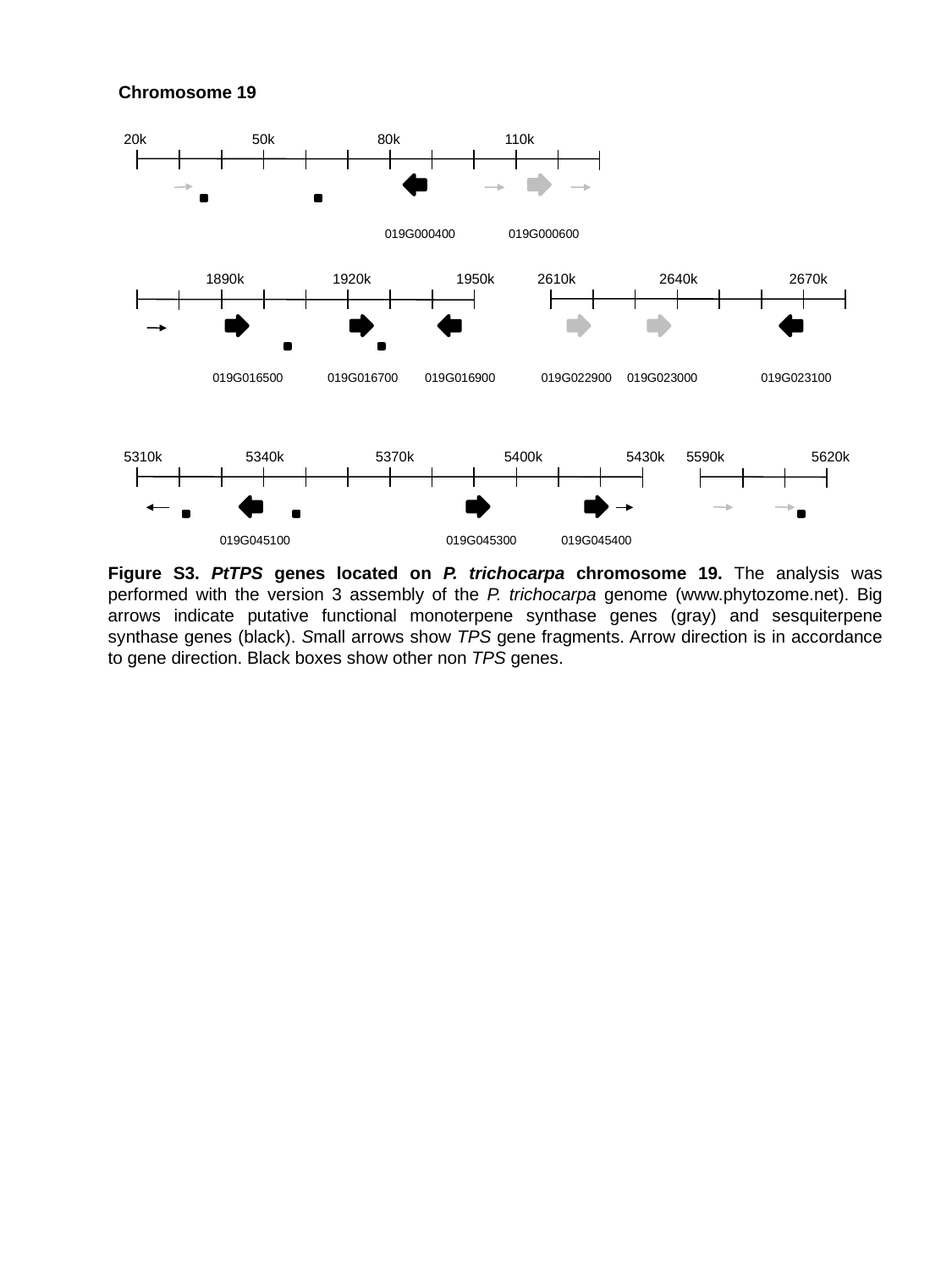

Chromosome 19
110k
80k
20k
50k
019G000400
019G000600
1950k
1890k
1920k
019G016500
019G016700
019G016900
2610k
2640k
2670k
019G022900
019G023000
019G023100
5370k
5430k
5400k
5310k
5340k
019G045100
019G045300
019G045400
5590k
5620k
Figure S3. PtTPS genes located on P. trichocarpa chromosome 19. The analysis was performed with the version 3 assembly of the P. trichocarpa genome (www.phytozome.net). Big arrows indicate putative functional monoterpene synthase genes (gray) and sesquiterpene synthase genes (black). Small arrows show TPS gene fragments. Arrow direction is in accordance to gene direction. Black boxes show other non TPS genes.

## Slide 4
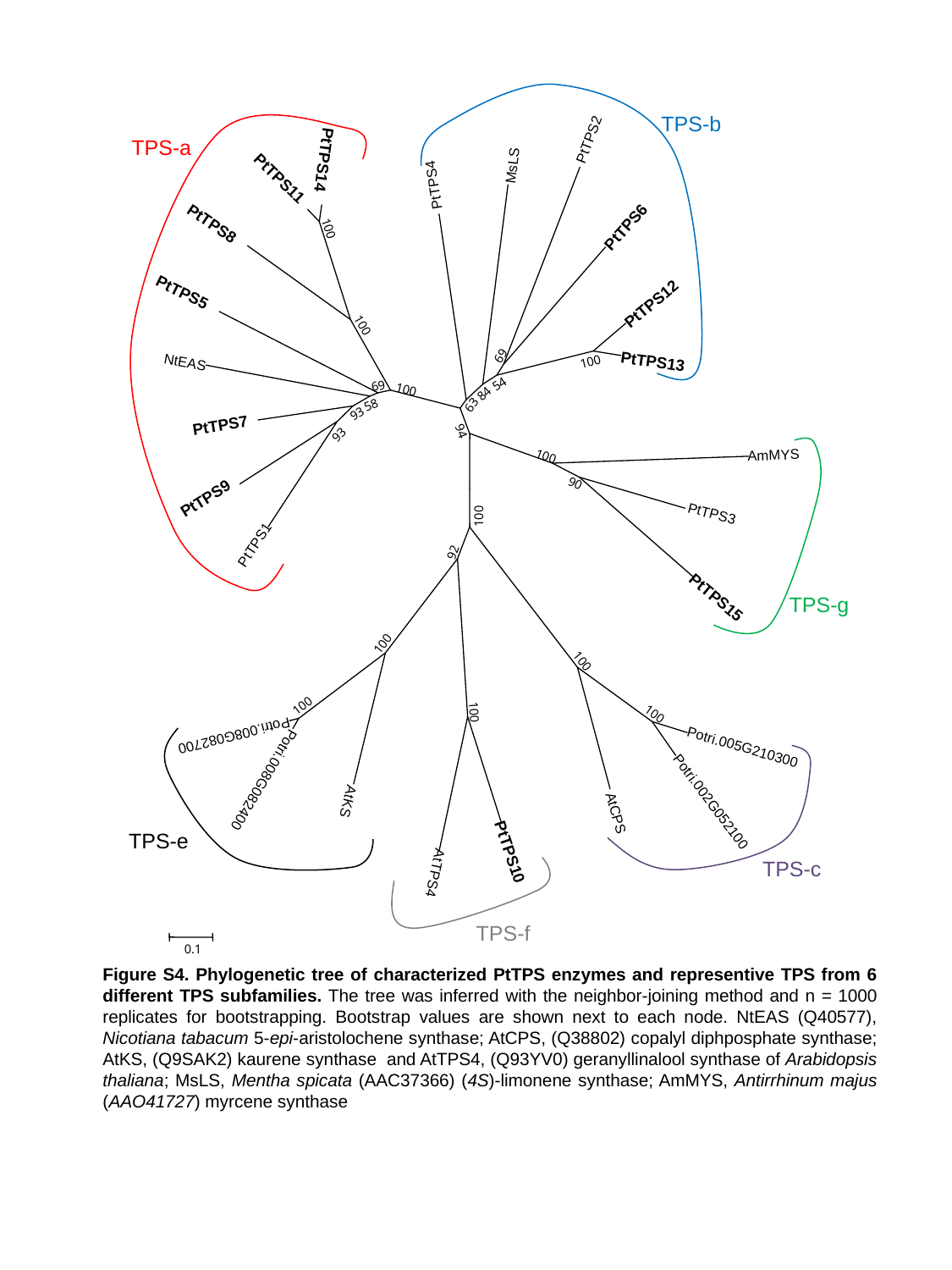

TPS-b
PtTPS2
PtTPS14
MsLS
PtTPS11
PtTPS4
PtTPS8
PtTPS6
100
PtTPS5
PtTPS12
100
69
PtTPS13
NtEAS
100
54
69
100
84
63
58
93
PtTPS7
94
93
AmMYS
100
90
PtTPS9
PtTPS3
100
PtTPS1
92
PtTPS15
100
100
100
100
100
Potri.008G082700
Potri.005G210300
Potri.008G082400
Potri.002G052100
AtKS
AtCPS
PtTPS10
AtTPS4
0.1
TPS-a
TPS-e
TPS-f
TPS-g
TPS-c
Figure S4. Phylogenetic tree of characterized PtTPS enzymes and representive TPS from 6 different TPS subfamilies. The tree was inferred with the neighbor-joining method and n = 1000 replicates for bootstrapping. Bootstrap values are shown next to each node. NtEAS (Q40577), Nicotiana tabacum 5-epi-aristolochene synthase; AtCPS, (Q38802) copalyl diphposphate synthase; AtKS, (Q9SAK2) kaurene synthase and AtTPS4, (Q93YV0) geranyllinalool synthase of Arabidopsis thaliana; MsLS, Mentha spicata (AAC37366) (4S)-limonene synthase; AmMYS, Antirrhinum majus (AAO41727) myrcene synthase

## Slide 5
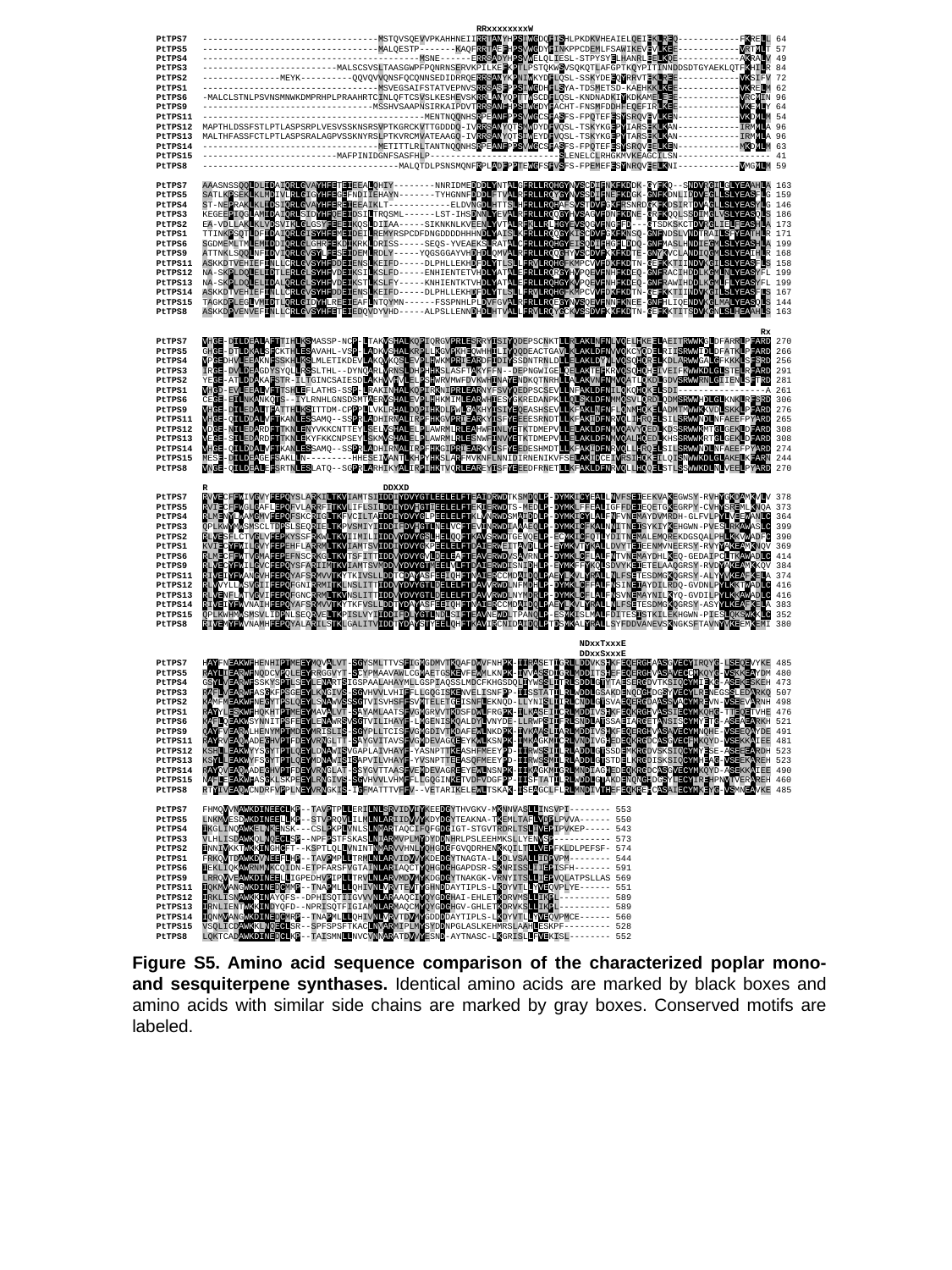

Figure S5. Amino acid sequence comparison of the characterized poplar mono- and sesquiterpene synthases. Identical amino acids are marked by black boxes and amino acids with similar side chains are marked by gray boxes. Conserved motifs are labeled.

## Slide 6
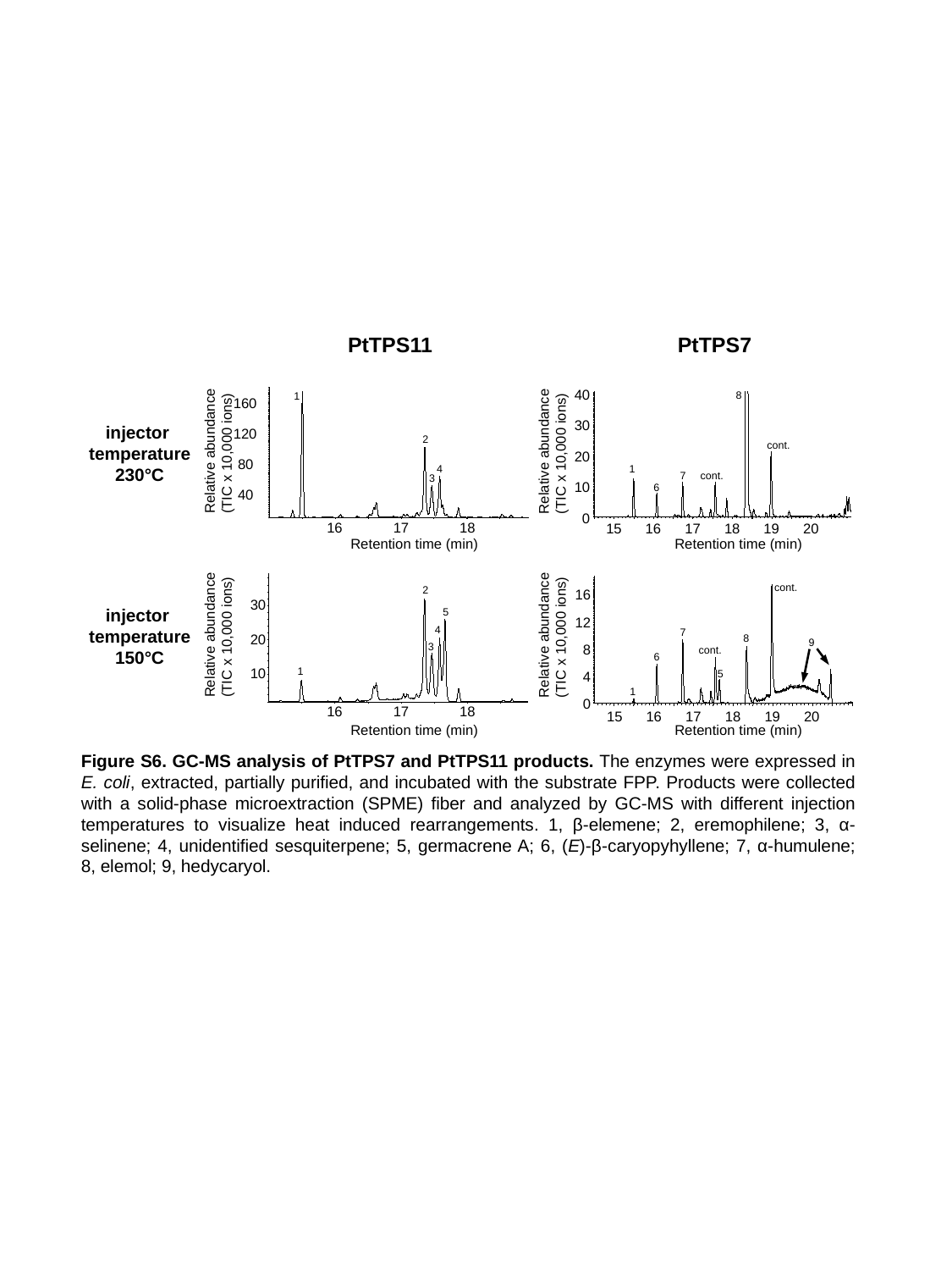

PtTPS11
PtTPS7
8
40
30
20
10
0
15
16
17
18
19
20
cont.
1
7
cont.
6
1
2
4
3
16
17
18
Retention time (min)
160
injector
temperature
230°C
120
Relative abundance
(TIC x 10,000 ions)
Relative abundance
(TIC x 10,000 ions)
80
40
Retention time (min)
2
30
5
4
20
3
1
10
16
17
18
Retention time (min)
cont.
16
12
7
8
9
cont.
8
6
5
4
1
0
15
16
17
18
19
20
injector
temperature
150°C
Relative abundance
(TIC x 10,000 ions)
Relative abundance
(TIC x 10,000 ions)
Retention time (min)
Figure S6. GC-MS analysis of PtTPS7 and PtTPS11 products. The enzymes were expressed in E. coli, extracted, partially purified, and incubated with the substrate FPP. Products were collected with a solid-phase microextraction (SPME) fiber and analyzed by GC-MS with different injection temperatures to visualize heat induced rearrangements. 1, β-elemene; 2, eremophilene; 3, α-selinene; 4, unidentified sesquiterpene; 5, germacrene A; 6, (E)-β-caryopyhyllene; 7, α-humulene; 8, elemol; 9, hedycaryol.

## Slide 7
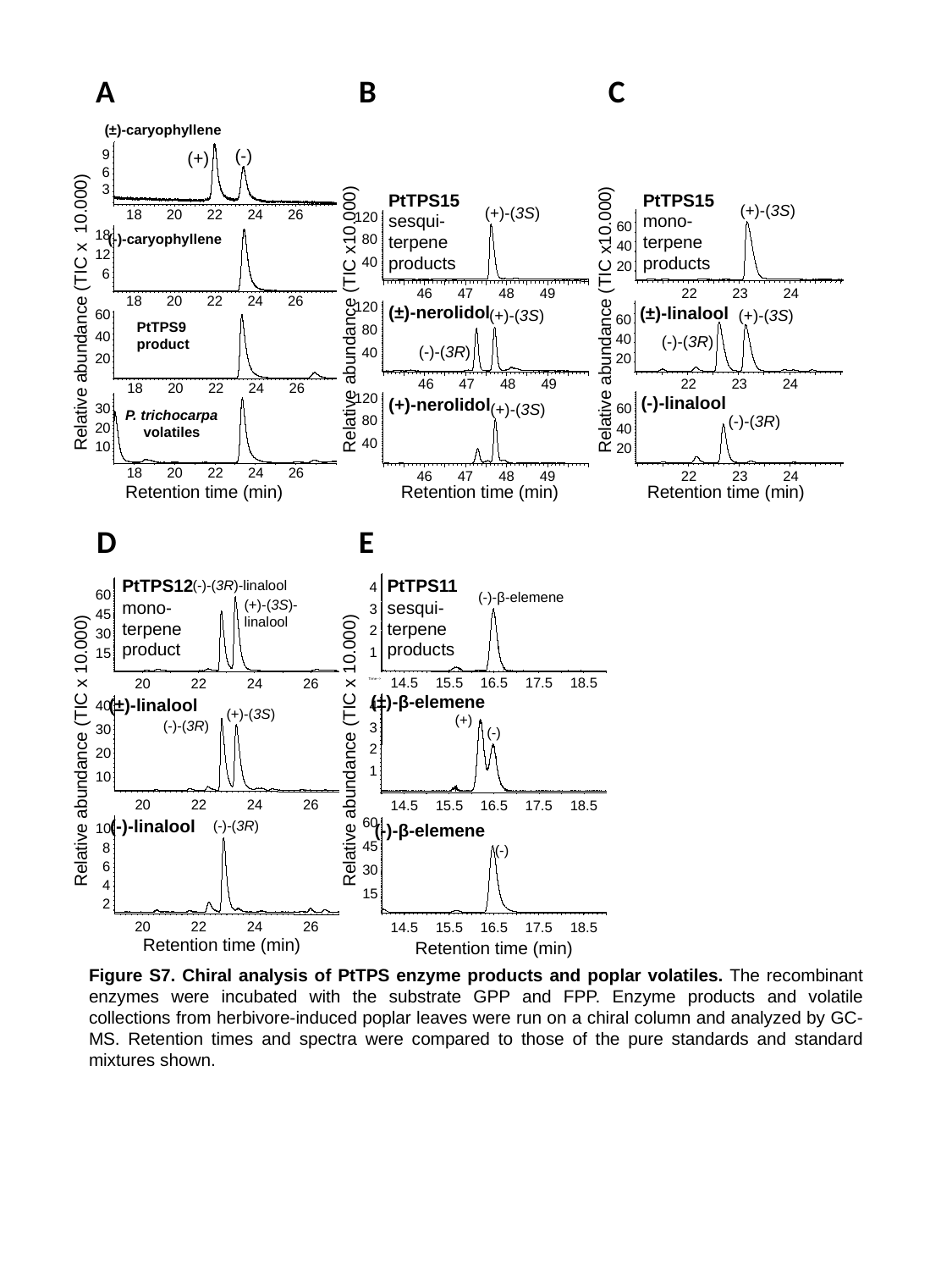

B
A
C
(±)-caryophyllene
(-)
(+)
9
6
3
18
20
22
24
26
(-)-caryophyllene
18
12
6
Relative abundance (TIC x 10.000)
18
20
22
24
26
60
PtTPS9
product
40
20
18
20
22
24
26
30
P. trichocarpa
volatiles
20
10
18
20
22
24
26
Retention time (min)
PtTPS15
sesqui-terpene products
PtTPS15
mono-terpene products
(+)-(3S)
60
40
20
22
23
24
(+)-(3S)
120
80
40
46
47
48
49
(±)-nerolidol
(±)-linalool
120
(+)-(3S)
(+)-(3S)
Relative abundance (TIC x10.000)
Relative abundance (TIC x10.000)
60
80
(-)-(3R)
40
(-)-(3R)
40
20
22
23
24
46
47
48
49
(-)-linalool
(+)-nerolidol
120
(+)-(3S)
60
(-)-(3R)
80
40
40
20
22
23
24
46
47
48
49
Retention time (min)
Retention time (min)
PtTPS12
mono-
terpene
product
PtTPS11
sesqui-
terpene
products
(-)-(3R)-linalool
4
(-)-β-elemene
60
(+)-(3S)-
linalool
3
45
2
30
1
15
14.5
15.5
16.5
17.5
18.5
20
22
24
26
Time-->
(±)-β-elemene
(±)-linalool
40
30
20
10
20
22
24
26
4
(+)-(3S)
(+)
(-)-(3R)
(-)
3
Relative abundance (TIC x 10.000)
Relative abundance (TIC x 10.000)
2
1
14.5
15.5
16.5
17.5
18.5
(-)-linalool
(-)-(3R)
(-)-β-elemene
60
10
8
6
4
2
20
22
24
26
(-)
45
30
15
14.5
15.5
16.5
17.5
18.5
Retention time (min)
Retention time (min)
D
E
Figure S7. Chiral analysis of PtTPS enzyme products and poplar volatiles. The recombinant enzymes were incubated with the substrate GPP and FPP. Enzyme products and volatile collections from herbivore-induced poplar leaves were run on a chiral column and analyzed by GC-MS. Retention times and spectra were compared to those of the pure standards and standard mixtures shown.

## Slide 8
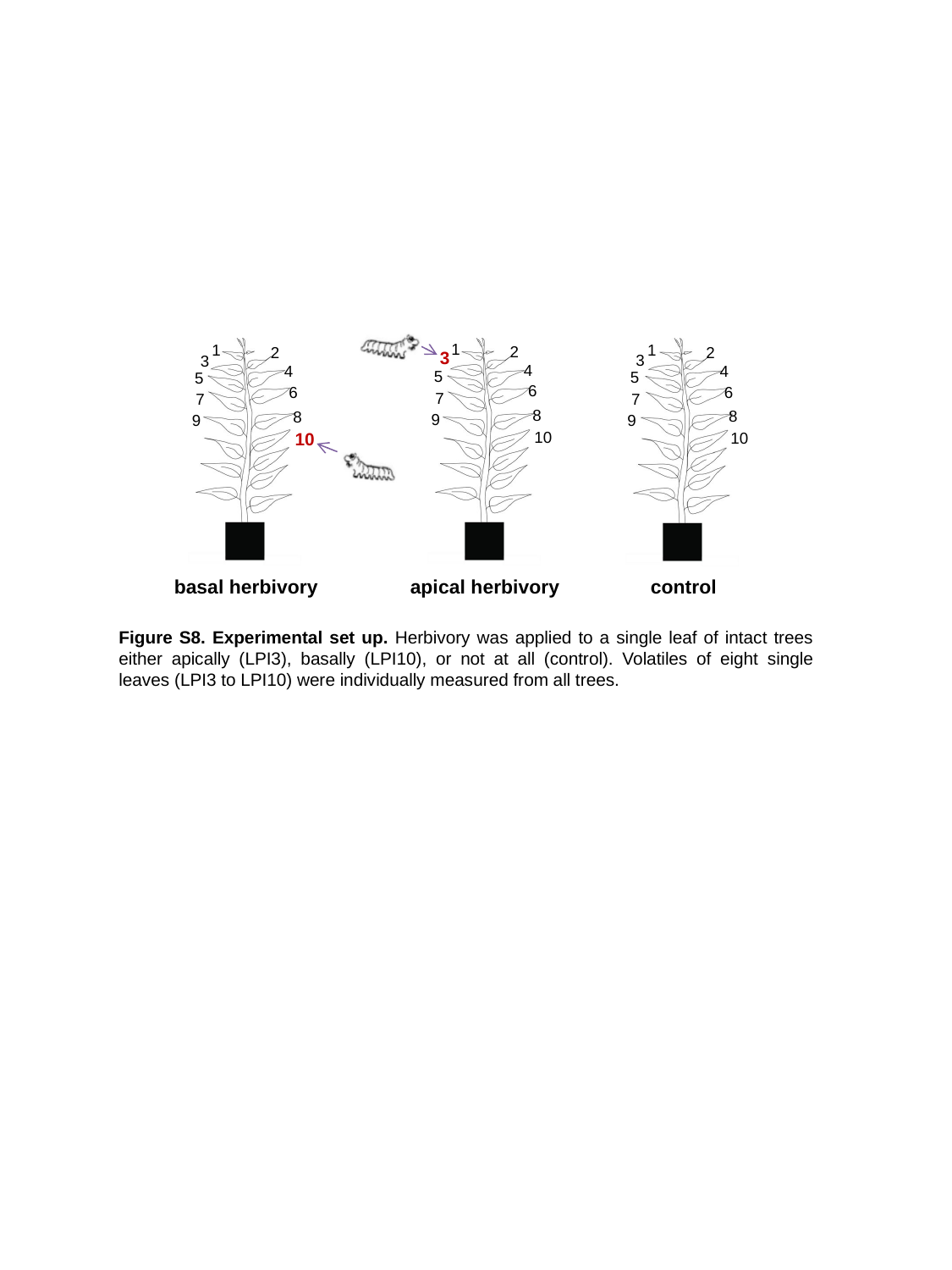

1
1
1
2
2
2
3
3
3
4
4
4
5
5
5
6
6
6
7
7
7
8
8
8
9
9
9
10
10
10
basal herbivory
apical herbivory
control
Figure S8. Experimental set up. Herbivory was applied to a single leaf of intact trees either apically (LPI3), basally (LPI10), or not at all (control). Volatiles of eight single leaves (LPI3 to LPI10) were individually measured from all trees.

## Slide 9
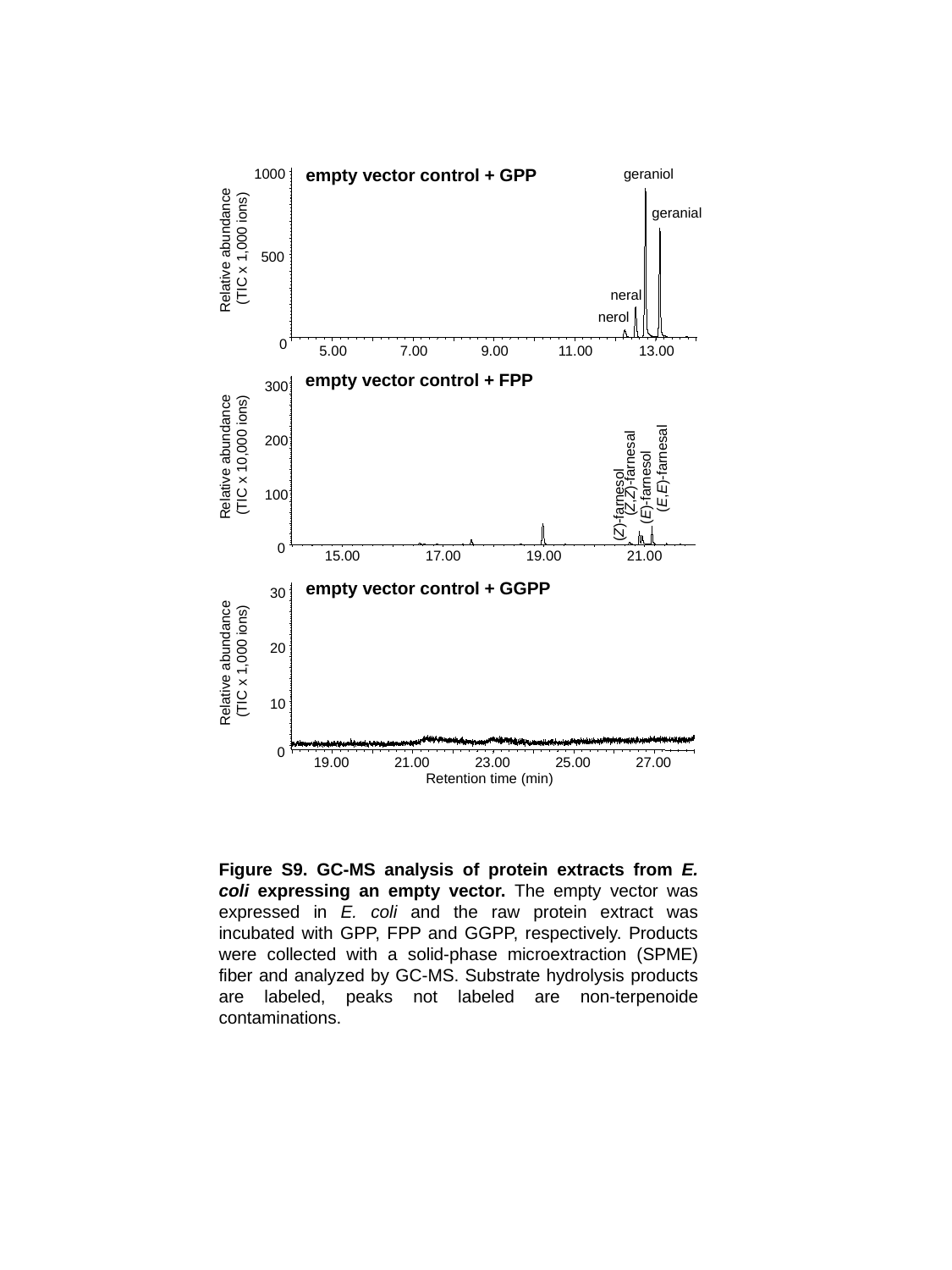

empty vector control + GPP
geraniol
1000
geranial
Relative abundance
 (TIC x 1,000 ions)
500
neral
nerol
0
5.00
7.00
9.00
11.00
13.00
empty vector control + FPP
300
200
Relative abundance
 (TIC x 10,000 ions)
(E,E)-farnesal
(Z,Z)-farnesal
(E)-farnesol
(Z)-farnesol
100
0
15.00
17.00
19.00
21.00
empty vector control + GGPP
30
20
10
0
19.00
21.00
23.00
25.00
27.00
Relative abundance
 (TIC x 1,000 ions)
Retention time (min)
Figure S9. GC-MS analysis of protein extracts from E. coli expressing an empty vector. The empty vector was expressed in E. coli and the raw protein extract was incubated with GPP, FPP and GGPP, respectively. Products were collected with a solid-phase microextraction (SPME) fiber and analyzed by GC-MS. Substrate hydrolysis products are labeled, peaks not labeled are non-terpenoide contaminations.
