# Supplementary material for: Terpene synthases and their contribution to herbivore-induced volatile emission in western balsam poplar (Populus trichocarpa)
Source: BMC Plant Biol. 2014 Oct 11;14:270. doi: 10.1186/s12870-014-0270-y (PMC4197230; doi:10.1186/s12870-014-0270-y)
Supplement: Additional file 2: — This file contains 10 supplemental tables. [file 12870_2014_270_MOESM2_ESM.docx]

**Table S1. Terpene synthase features.** Predicted nucleotide (nt) and amino acid (aa) length, terpene synthase type, signal peptide (TargetP 1.1) length and conserved motifs of poplar TPS enzymes are listed. MT, monoterpene synthase ; ST, sesquiterpene synthase ; DT, diterpene synthase.

| TPS | nt | Aa | type | RR(x)_8_W | RXR | DDXXD | NSE/DTE | TargetP 1.1 |
| --- | --- | --- | --- | --- | --- | --- | --- | --- |
| PtTPS5 | 1650 | 550 | ST | RR(x)_8_W | RXR | DDXXD | DDxxSxxxE | - |
| PtTPS6 | 1773 | **591** | **MT** | RR(x)_8_W | RXR | DDXXD | NDxxTxxxE | 27 |
| PtTPS7 | 1659 | 553 | ST | RR(x)_8_W | RXR | DDXXD | DDxxSxxxE | - |
| PtTPS9 | 1707 | 569 | ST | RR(x)_8_W | RXR | DDXXD | DDxxSxxxE | - |
| PtTPS8 | 1656 | 552 | ST | R**P**(x)_8_W | RXR | DDXXD | NDxxTxxxE | - |
| PtTPS10 | 2499 | **833** | **DT** | - | RXK | DDXXD | NDxxSxxxE | - |
| PtTPS11 | 1653 | 551 | ST | R**P**(x)_8_W | RXR | DDXXD | NDxx**G**xxxE | - |
| PtTPS12 | 1704 | **568** | **MT** | RR(x)_8_W | RXR | DDXXD | DDxxTxxxE | 23 |
| PtTPS13 | 1767 | **589** | **MT** | RR(x)_8_W | RXR | DDXXD | DDxxTxxxE | 40 |
| PtTPS14 | 1680 | 560 | ST | R**P**(x)_8_W | RXR | DDXXD | NDxx**G**xxxE | - |
| PtTPS15 | 1581 | **527** | **MT** | **-** | RX**Q** | DDXXD | DDxxTxxxE | - |

**Supplemental table 2. Statistical analysis of *TPS* gene expression in individual leaves of *P. trichocarpa*.** Leaves treated with herbivores or taken from a tree which received herbivory were compared with the respective leaves from control trees using the student´s t-test. t-values and p-values are shown. ha, herbivory apical on LPI3; hb; herbivory basal on LPI10. * was log transformed for statistical analysis.

| ***TPS*** | **leaf** | **transformation** | **t value** | **p value** |
| --- | --- | --- | --- | --- |
| *PtTPS6* | hb | 10 | 3.120 | **0.014** |
|  | hb* | 9 | -2.430 | **0.041** |
|  | hb* | 5 | -1.192 | 0.268 |
|  | ha* | 3 | 5.764 | **< 0.001** |
|  | ha* | 4 | -0.00551 | 0.996 |
|  | ha | 8 | -0.964 | 0.363 |
| *PtTPS9* | hb* | 10 | -1.737 | 0.121 |
|  | hb | 9 | -0.675 | 0.519 |
|  | hb | 5 | -0.436 | 0.674 |
|  | ha | 3 | 3.101 | **0.015** |
|  | ha | 4 | 1.449 | 0.191 |
|  | ha | 8 | -0.594 | 0.569 |
| *PtTPS5* | ha | 3 | 1.776 | 0.114 |
| *PtTPS7* | ha* | 3 | 3.602 | **0.007** |
| *PtTPS10* | ha* | 3 | 4.389 | **0.002** |
| *PtTPS11/14* | ha* | 3 | 1.322 | 0.223 |
| *PtTPS12* | ha* | 3 | 2.374 | **0.045** |
| *PtTPS13* | ha* | 3 | 4.383 | **0.002** |
| *PtTPS15* | ha* | 3 | 3.906 | **0.005** |

**Supplemental table 3. ΔCq values of different poplar terpenes synthases.** QRT-PCR analysis was performed with cDNA attained from herbivore induced *P. trichocarpa* leaves. Means (n = 5) and standard errors (SE) are shown.

| ***TPS* gene** | **ΔCq values** | **SE** |
| --- | --- | --- |
| *PtTPS5* | 9.67 | 0.78 |
| *PtTPS6* | 3.66 | 0.67 |
| *PtTPS7* | 13.17 | 1.10 |
| *PtTPS9* | 4.96 | 0.35 |
| *PtTPS10* | 11.13 | 0.91 |
| *PtTPS11/14* | 15.19 | 0.66 |
| *PtTPS12* | 17.44 | 0.01 |
| *PtTPS13* | 13.19 | 0.80 |
| *PtTPS15* | 9.25 | 0.42 |

## Supplemental table 4. Volatiles emitted from individual leaves of *P. trichocarpa* trees (ng g^-1^ fresh weight h^-1^).

Herbivory (h) was imposed by *L. dispar* larvae feeding on either (A) a basal leaf, number 10 (hb-10), (B) an apical leaf, number 3 (ha-3) or (C) not at all (control, ctr). Herbivore-damaged leaves are shaded in gray. Volatiles were separately collected from leaves 3 to 10 and analyzed using GC-MS and GC-FID. Means and standard errors (SE) are shown (n = 5). RT, retention time.

**(A)**

| **RT** |  | **hb-3** | | SE | **hb -4** | SE | | **hb -5** | SE | **hb -6** | SE | **hb -7** | | | SE | **hb -8** | | SE | **hb -9** | SE | **hb -10** | SE | |
| --- | --- | --- | --- | --- | --- | --- | --- | --- | --- | --- | --- | --- | --- | --- | --- | --- | --- | --- | --- | --- | --- | --- | --- |
|  | **Unidentified** |  | |  |  |  | |  |  |  |  |  | | |  |  | |  |  |  |  |  | |
| 9.20 | C1 | 3.38 | | 0.33 | 3.67 | 0.64 | | 2.42 | 0.17 | 3.02 | 0.48 | 3.23 | | | 0.46 | 3.06 | | 0.43 | 4.10 | 0.40 | 6.47 | 2.72 | |
| 9.30 | C2 | 1.40 | | 0.14 | 1.53 | 0.28 | | 0.99 | 0.08 | 1.25 | 0.21 | 1.34 | | | 0.21 | 1.27 | | 0.17 | 1.69 | 0.17 | 2.67 | 1.13 | |
| 10.17 | C3 | 0.76 | | 0.08 | 0.81 | 0.14 | | 0.52 | 0.04 | 0.68 | 0.11 | 0.72 | | | 0.10 | 0.68 | | 0.10 | 0.94 | 0.08 | 1.71 | 0.50 | |
| 10.32 | C4 | 4.08 | | 0.41 | 4.51 | 0.78 | | 2.87 | 0.22 | 3.66 | 0.62 | 3.92 | | | 0.57 | 3.68 | | 0.54 | 4.99 | 0.46 | 7.93 | 3.17 | |
| 10.47 | C5 | 1.54 | | 0.18 | 1.80 | 0.34 | | 1.07 | 0.11 | 1.44 | 0.24 | 1.51 | | | 0.28 | 1.42 | | 0.24 | 2.01 | 0.17 | 2.99 | 1.30 | |
| 10.7 | C6 | 1.00 | | 0.10 | 1.14 | 0.23 | | 0.62 | 0.05 | 0.79 | 0.14 | 0.86 | | | 0.12 | 0.79 | | 0.11 | 1.26 | 0.13 | 2.13 | 0.58 | |
| 11.25 | C7 | 0.00 | | 0.00 | 0.00 | 0.00 | | 0.00 | 0.00 | 0.00 | 0.00 | 0.00 | | | 0.00 | 0.00 | | 0.00 | 0.00 | 0.00 | 12.98 | 8.95 | |
| 14.7 | C9 | 0.00 | 0.00 | | 0.00 | 0.00 | | 0.00 | 0.00 | 0.00 | 0.00 | 0.00 | | 0.00 | | 0.00 | 0.00 | | 0.00 | 0.00 | 3.04 | 2.26 | |
| 18.09 | C10 | 0.00 | 0.00 | | 0.09 | 0.09 | | 0.00 | 0.00 | 0.00 | 0.00 | 0.00 | | 0.00 | | 0.00 | 0.00 | | 0.00 | 0.00 | 1.65 | 1.09 | |
| 24.04 | C11 | 0.00 | 0.00 | | 0.00 | 0.00 | | 0.00 | 0.00 | 0.00 | 0.00 | 0.00 | | 0.00 | | 0.00 | 0.00 | | 0.00 | 0.00 | 2.75 | 0.92 | |
| 29.63 | C15 | 0.00 | | 0.00 | 0.00 | 0.00 | 0.00 | | 0.00 | 0.00 | 0.00 | 0.00 | | 0.00 | | 0.00 | | 0.00 | 0.00 | 0.00 | 0.34 | 0.23 | |
| 31.14 | C16 | 0.47 | | 0.15 | 0.38 | 0.18 | 0.41 | | 0.18 | 0.67 | 0.11 | 0.43 | | 0.16 | | 0.71 | | 0.51 | 0.68 | 0.31 | 1.25 | 0.51 | |
| 31.25 | C17 | 0.61 | | 0.19 | 0.49 | 0.23 | 0.59 | | 0.26 | 0.94 | 0.22 | 0.53 | | 0.19 | | 0.94 | | 0.69 | 1.05 | 0.37 | 0.99 | 0.26 | |
| 31.58 | C18 | 0.00 | | 0.00 | 0.06 | 0.06 | 0.00 | | 0.00 | 0.00 | 0.00 | 0.00 | | 0.00 | | 0.00 | | 0.00 | 0.00 | 0.00 | 14.97 | 8.48 | |
| 33.65 | C19 | 0.00 | | 0.00 | 0.00 | 0.00 | 0.00 | | 0.00 | 0.00 | 0.00 | 0.00 | | 0.00 | | 0.00 | | 0.00 | 0.00 | 0.00 | 0.00 | 0.00 | |
| 33.31 | C20 | 0.00 | | 0.00 | 0.00 | 0.00 | 0.00 | | 0.00 | 0.00 | 0.00 | 0.00 | | 0.00 | | 0.00 | | 0.00 | 0.00 | 0.00 | 3.34 | 2.27 | |
|  |  |  | |  |  |  |  | |  |  |  |  | |  | |  | |  |  |  |  |  | |
|  | **Aldehydes** |  | |  |  |  |  | |  |  |  |  | |  | |  | |  |  |  |  |  | |
| 9.86 | hexanal | 0.00 | | 0.00 | 0.00 | 0.00 | 0.00 | | 0.00 | 0.09 | 0.09 | 0.00 | | 0.00 | | 0.00 | | 0.00 | 0.00 | 0.00 | 1.94 | 0.51 | |
| 13.22 | *trans*-2-hexenal | 0.00 | | 0.00 | 0.00 | 0.00 | 0.00 | | 0.00 | 0.00 | 0.00 | 0.00 | | 0.00 | | 0.00 | | 0.00 | 0.00 | 0.00 | 14.94 | 6.18 | |
| 16.41 | 6-methyl-5-hepten-2-one | traces | | | | | | | | | | | | | | | | | | | | | |
| 17.93 | nonanal | 1.20 | | 0.19 | 1.54 | 0.32 | 1.00 | | 0.15 | 1.88 | 0.44 | 1.66 | 0.40 | | | 1.35 | | 0.42 | 1.50 | 0.22 | 16.01 | 12.53 | |
| 21.17 | benzaldehyde | 0.48 | | 0.13 | 0.28 | 0.17 | 0.00 | | 0.00 | 0.21 | 0.14 | 0.11 | 0.11 | | | 0.00 | | 0.00 | 0.00 | 0.00 | 0.92 | 0.36 | |
| 24.15 | benzenacetaldehyde | 0.00 | | 0.00 | 0.00 | 0.00 | 0.00 | | 0.00 | 0.00 | 0.00 | 0.00 | 0.00 | | | 0.00 | | 0.00 | 0.00 | 0.00 | 2.91 | 1.87 | |
|  |  |  | |  |  |  |  | |  |  |  |  |  | | |  | |  |  |  |  |  | |
|  | **Nitrogen containing compounds** | | | | | |  | |  |  |  |  |  | | |  | |  |  |  |  |  | |
| 9.93 | 2-methylbutyronitrile | 0.00 | | 0.00 | 0.00 | 0.00 | 0.00 | | 0.00 | 0.00 | 0.00 | 0.00 | 0.00 | | | 0.00 | | 0.00 | 0.00 | 0.00 | 1.98 | 1.29 | |
| 10.81 | 3-methylbutyronitrile | 0.00 | | 0.00 | 0.00 | 0.00 | 0.00 | | 0.00 | 0.00 | 0.00 | 0.00 | 0.00 | | | 0.00 | | 0.00 | 0.00 | 0.00 | 4.33 | 3.26 | |
| 17.68 | (*E*)-isobutyraldoxime | 0.00 | | 0.00 | 0.00 | 0.00 | 0.00 | | 0.00 | 0.00 | 0.00 | 0.00 | 0.00 | | | 0.00 | | 0.00 | 0.00 | 0.00 | 1.19 | 0.47 | |
| 18.17 | (*Z*)-isobutyraldoxime | 0.00 | | 0.00 | 0.00 | 0.00 | 0.00 | | 0.00 | 0.00 | 0.00 | 0.00 | 0.00 | | | 0.00 | | 0.00 | 0.00 | 0.00 | 0.91 | 0.23 | |
| 20.40 | (*E*)-2-methyl-butyraldoxime | 0.00 | | 0.00 | 0.00 | 0.00 | 0.00 | | 0.00 | 0.00 | 0.00 | 0.00 | 0.00 | | | 0.00 | | 0.00 | 0.00 | 0.00 | 18.17 | 4.74 | |
| 20.55 | (*E*)-3-methyl-butyraldoxime | 0.00 | | 0.00 | 0.00 | 0.00 | 0.00 | | 0.00 | 0.00 | 0.00 | 0.00 | 0.00 | | | 0.00 | | 0.00 | 0.00 | 0.00 | 14.10 | 3.39 | |
| 20.86 | (*Z*)-2-methyl-butyraldoxime | 0.00 | | 0.00 | 0.00 | 0.00 | 0.00 | | 0.00 | 0.00 | 0.00 | 0.00 | 0.00 | | | 0.00 | | 0.00 | 0.00 | 0.00 | 6.42 | 1.41 | |
| 21.48 | (*Z*)-3-methyl-butyraldoxime | 0.00 | | 0.00 | 0.00 | 0.00 | 0.00 | | 0.00 | 0.00 | 0.00 | 0.00 | 0.00 | | | 0.00 | | 0.00 | 0.00 | 0.00 | 8.65 | 2.52 | |
| 30.30 | benzyl cyanide | 0.00 | | 0.00 | 0.00 | 0.00 | 0.00 | | 0.00 | 0.00 | 0.00 | 0.00 | 0.00 | | | 0.00 | | 0.00 | 0.00 | 0.00 | 55.64 | 12.31 | |
| 34.06 | phenylnitroethane | 0.00 | | 0.00 | 0.00 | 0.00 | 0.00 | | 0.00 | 0.00 | 0.00 | 0.00 | 0.00 | | | 0.00 | | 0.00 | 0.00 | 0.00 | 1.33 | 0.31 | |
| 37.16 | (*E*)-phenylacetaldoxime | 0.00 | | 0.00 | 0.00 | 0.00 | 0.00 | | 0.00 | 0.00 | 0.00 | 0.00 | 0.00 | | | 0.00 | | 0.00 | 0.00 | 0.00 | 0.29 | 0.07 | |
| 37.88 | (*Z*)-phenylacetaldoxime | 0.00 | | 0.00 | 0.00 | 0.00 | 0.00 | | 0.00 | 0.00 | 0.00 | 0.00 | 0.00 | | | 0.00 | | 0.00 | 0.00 | 0.00 | 0.41 | 0.11 | |
| 39.90 | indole | 1.10 | | 0.54 | 0.72 | 0.52 | 0.32 | | 0.32 | 0.00 | 0.00 | 0.00 | 0.00 | | | 0.18 | | 0.18 | 0.00 | 0.00 | 13.91 | 5.99 | |
|  |  |  | |  |  |  |  | |  |  |  |  |  | | |  | |  |  |  |  |  | |
|  | **Monoterpenes** |  | |  |  |  |  | |  |  |  |  |  | | |  | |  |  |  |  |  | |
| 8.73 | α-pinene | 0.00 | | 0.00 | 0.00 | 0.00 | 0.00 | | 0.00 | 0.00 | 0.00 | 0.00 | 0.00 | | | 0.00 | | 0.00 | 0.00 | 0.00 | traces | | |
| 11.88 | myrcene | 0.22 | | 0.13 | 0.10 | 0.10 | 0.18 | | 0.13 | 0.18 | 0.12 | 0.08 | 0.08 | | | 0.00 | | 0.00 | 0.00 | 0.00 | 0.75 | 0.26 | |
| 12.78 | limonene | 0.00 | | 0.00 | 0.00 | 0.00 | 0.00 | | 0.00 | 0.07 | 0.07 | 0.00 | 0.00 | | | 0.00 | | 0.00 | 0.00 | 0.00 | 0.54 | 0.30 | |
| 13.13 | 1,8-cineole | 0.00 | | 0.00 | 0.00 | 0.00 | 0.00 | | 0.00 | 0.00 | 0.00 | 0.00 | 0.00 | | | 0.00 | | 0.00 | 0.00 | 0.00 | traces | | |
| 13.70 | (*Z*)-β-ocimene | 0.11 | | 0.11 | 0.07 | 0.07 | 0.18 | | 0.11 | 0.08 | 0.08 | 0.00 | 0.00 | | | 0.00 | | 0.00 | 0.00 | 0.00 | 5.45 | 1.55 | |
| 14.16 | (*E*)- β-ocimene | 3.10 | | 0.73 | 1.45 | 0.54 | 2.42 | | 1.23 | 1.46 | 0.71 | 0.88 | 0.20 | | | 0.75 | | 0.39 | 0.16 | 0.16 | 117.1 | 38.51 | |
| 17.35 | alloocimene | 0.00 | | 0.00 | 0.00 | 0.00 | 0.00 | | 0.00 | 0.00 | 0.00 | 0.00 | 0.00 | | | 0.00 | | 0.00 | 0.00 | 0.00 | 0.09 | 0.09 | |
| 21.75 | linalool | 5.71 | | 0.85 | 2.86 | 1.13 | 1.59 | | 0.45 | 1.21 | 0.29 | 1.20 | 0.28 | | | 0.60 | | 0.31 | 0.18 | 0.18 | 1.08 | 0.25 | |
| 20.30 | (*E*)-epoxy-ocimene | 0.00 | | 0.00 | 0.00 | 0.00 | 0.00 | | 0.00 | 0.00 | 0.00 | 0.00 | 0.00 | | | 0.00 | | 0.00 | 0.00 | 0.00 | 1.84 | 0.59 | |
| 26.87 | citronellol | 0.00 | | 0.00 | 0.00 | 0.00 | 0.00 | | 0.00 | 0.00 | 0.00 | 0.00 | 0.00 | | | 0.00 | | 0.00 | 0.00 | 0.00 | traces | | |
|  |  |  | |  |  |  |  | |  |  |  |  |  | | |  | |  |  |  |  |  | |
|  | **Homoterpenes** |  | |  |  |  |  | |  |  |  |  |  | | |  | |  |  |  |  |  | |
| 15.66 | DMNT | 0.67 | | 0.40 | 0.37 | 0.25 | 0.64 | | 0.27 | 0.36 | 0.23 | 0.39 | 0.17 | | | 0.33 | | 0.23 | 0.00 | 0.00 | 1.93 | 1.23 | |
| 27.86 | TMTT | 0.00 | | 0.00 | 0.00 | 0.00 | 0.00 | | 0.00 | 0.00 | 0.00 | 0.00 | 0.00 | | | 0.00 | | 0.00 | 0.00 | 0.00 | 0.56 | 0.24 | |
|  |  |  | |  |  |  |  | |  |  |  |  |  | | |  | |  |  |  |  |  | |
|  | **Alcohols** |  | |  |  |  |  | |  |  |  |  |  | | |  | |  |  |  |  |  | |
| 12.90 | 2/3-methylbutanol | 0.00 | | 0.00 | 0.00 | 0.00 | 0.00 | | 0.00 | 0.00 | 0.00 | 0.00 | 0.00 | | | 0.00 | | 0.00 | 0.00 | 0.00 | traces | | |
| 16.82 | 1-hexanol | 0.00 | | 0.00 | 0.00 | 0.00 | 0.00 | | 0.00 | 0.09 | 0.09 | 0.00 | 0.00 | | | 0.00 | | 0.00 | 0.00 | 0.00 | 47.39 | 28.75 | |
| 17.62 | (*Z*)-3-hexenol | 2.55 | | 0.51 | 4.19 | 1.96 | 1.91 | | 0.92 | 1.90 | 0.46 | 1.65 | 0.54 | | | 1.01 | | 0.30 | 0.54 | 0.29 | 82.64 | 36.83 | |
| 18.19 | (*Z*)-2-hexenol | 0.00 | | 0.00 | 0.00 | 0.00 | 0.00 | | 0.00 | 0.00 | 0.00 | 0.00 | 0.00 | | | 0.00 | | 0.00 | 0.00 | 0.00 | 19.16 | 14.68 | |
| 22.04 | 1-octanol | 0.58 | | 0.18 | 0.53 | 0.24 | 0.68 | | 0.22 | 0.86 | 0.15 | 0.77 | 0.07 | | | 0.66 | | 0.43 | 0.66 | 0.21 | 8.20 | 6.62 | |
| 24.50 | nonanol | 1.16 | | 0.43 | 1.28 | 0.45 | 1.40 | | 0.37 | 1.74 | 0.36 | 1.57 | 0.16 | | | 1.60 | | 0.70 | 1.51 | 0.51 | 4.52 | 1.32 | |
| 29.30 | benzyl alcohol | 1.66 | | 0.32 | 0.85 | 0.30 | 0.58 | | 0.22 | 0.47 | 0.15 | 0.46 | 0.04 | | | 0.29 | | 0.22 | 0.00 | 0.00 | 12.14 | 7.87 | |
| 29.98 | phenylethyl alcohol | 3.37 | | 0.76 | 1.77 | 1.05 | 0.84 | | 0.46 | 0.88 | 0.51 | 0.82 | 0.73 | | | 0.05 | | 0.05 | 0.00 | 0.00 | 42.77 | 10.43 | |
| 31.14 | 1.2-(*E*)-cyclohexandiol | 0.00 | | 0.00 | 0.00 | 0.00 | 0.00 | | 0.00 | 0.00 | 0.00 | 0.00 | 0.00 | | | 0.00 | | 0.00 | 0.00 | 0.00 | 6.70 | 5.08 | |
|  |  |  | |  |  |  |  | |  |  |  |  |  | | |  | |  |  |  |  |  | |
|  | **Esters** |  | |  |  |  |  | |  |  |  |  |  | | |  | |  |  |  |  |  | |
| 15.87 | (*Z*)-3-hexenyl acetate | 1.44 | | 0.46 | 6.55 | 5.51 | 3.03 | | 1.92 | 2.19 | 0.78 | 1.30 | 0.66 | | | 1.31 | | 1.03 | 0.34 | 0.21 | 15.25 | 9.58 | |
| 19.67 | (*Z*)-3-hexenyl butyrate | 0.09 | | 0.09 | 0.06 | 0.06 | 0.20 | | 0.14 | 0.00 | 0.00 | 0.00 | 0.00 | | | 0.00 | | 0.00 | 0.00 | 0.00 | 1.78 | 1.44 | |
| 20.05 | (*Z*)-3-hexenyl-2-methyl butyrate | 0.17 | | 0.17 | 0.14 | 0.14 | 0.10 | | 0.10 | 0.10 | 0.10 | 0.00 | 0.00 | | | 0.00 | | 0.00 | 0.00 | 0.00 | 0.94 | 0.80 | |
| 27.16 | methyl salicylate | 1.05 | | 0.80 | 0.00 | 0.00 | 0.00 | | 0.00 | 0.00 | 0.00 | 0.00 | 0.00 | | | 0.00 | | 0.00 | 0.00 | 0.00 | 0.00 | 0.00 | |
| 27.97 | phenylethyl acetate | 0.00 | | 0.00 | 0.00 | 0.00 | 0.00 | | 0.00 | 0.00 | 0.00 | 0.00 | 0.00 | | | 0.00 | | 0.00 | 0.00 | 0.00 | 0.51 | 0.21 | |
| 34.28 | (*Z*)-3-hexenyl benzoate | 1.16 | | 0.38 | 0.33 | 0.20 | 0.40 | | 0.31 | 0.10 | 0.10 | 0.00 | 0.00 | | | 0.00 | | 0.00 | 0.00 | 0.00 | 2.09 | 1.58 | |
|  |  |  | |  |  |  |  | |  |  |  |  |  | | |  | |  |  |  |  |  | |
|  | **Sesquiterpenes** |  | |  |  |  |  | |  |  |  |  |  | | |  | |  |  |  |  |  | |
| 19.76 | α-cubebene | 0.00 | | 0.00 | 0.00 | 0.00 | 0.00 | | 0.00 | 0.00 | 0.00 | 0.00 | 0.00 | | | 0.00 | | 0.00 | 0.00 | 0.00 | 0.00 | 0.00 | |
| 20.63 | α-copaene | 0.00 | | 0.00 | 0.00 | 0.00 | 0.00 | | 0.00 | 0.00 | 0.00 | 0.00 | 0.00 | | | 0.00 | | 0.00 | 0.00 | 0.00 | traces | | |
| 23.20 | (*E*)-β-caryophyllene | 17.12 | | 3.94 | 15.39 | 6.37 | 19.66 | | 5.76 | 20.41 | 7.49 | 17.36 | 8.48 | | | 8.81 | | 3.55 | 7.11 | 4.20 | 16.78 | | 8.57 |
| 24.70 | (*E*) -β-farnesene | 0.00 | | 0.00 | 0.13 | 0.13 | 0.16 | | 0.16 | 0.26 | 0.17 | 0.11 | 0.11 | | | 0.00 | | 0.00 | 0.00 | 0.00 | 0.32 | | 0.14 |
| 24.90 | α-humulene | 0.73 | | 0.25 | 0.67 | 0.31 | 0.79 | | 0.34 | 0.80 | 0.50 | 0.79 | 0.55 | | | 0.37 | | 0.24 | 0.27 | 0.27 | 0.35 | | 0.23 |
| 25.80 | germacrene D | 0.00 | | 0.00 | 0.00 | 0.00 | 0.00 | | 0.00 | 0.00 | 0.00 | 0.00 | 0.00 | | | 0.00 | | 0.00 | 0.00 | 0.00 | 3.76 | | 1.63 |
| 26.03 | (*Z*.*E*)-α-farnesene | 2.41 | | 0.74 | 2.10 | 0.96 | 1.20 | | 0.59 | 0.67 | 0.33 | 1.16 | 0.40 | | | 0.95 | | 0.40 | 0.60 | 0.40 | 14.11 | | 4.05 |
| 26.57 | (*E*.*E*)-α-farnesene | 44.60 | | 8.59 | 49.62 | 22.91 | 34.19 | | 16.75 | 18.58 | 7.47 | 23.93 | 6.55 | | | 19.38 | | 7.26 | 15.19 | 7.00 | 309.0 | | 89.53 |
| 26.80 | δ-cadinene | 0.64 | | 0.17 | 0.39 | 0.39 | 0.12 | | 0.12 | 0.07 | 0.07 | 0.13 | 0.13 | | | 0.00 | | 0.00 | 0.00 | 0.00 | 1.00 | | 0.51 |
| 32.56 | nerolidol | 0.41 | | 0.25 | 0.07 | 0.07 | 0.17 | | 0.10 | 0.00 | 0.00 | 0.00 | 0.00 | | | 0.00 | | 0.00 | 0.00 | 0.00 | 0.00 | | 0.00 |

**(B)**

| **RT** |  | **ha-3** | | SE | | | **ha-4** | | | SE | | | **ha-5** | SE | **ha-6** | | | SE | **ha-7** | SE | **ha-8** | | | | SE | **ha-9** | | StE | **ha-10** | | SE |
| --- | --- | --- | --- | --- | --- | --- | --- | --- | --- | --- | --- | --- | --- | --- | --- | --- | --- | --- | --- | --- | --- | --- | --- | --- | --- | --- | --- | --- | --- | --- | --- |
|  | **Unidentified** |  | |  | | |  | | |  | | |  |  |  | | |  |  |  |  | | | |  |  | |  |  | |  |
| 9.20 | C1 | 3.77 | | 0.34 | | | 5.06 | | | 0.95 | | | 4.45 | 0.77 | 5.01 | | | 0.93 | 5.32 | 0.84 | 4.28 | | | | 0.94 | 9.18 | | 4.19 | 7.23 | | 2.12 |
| 9.30 | C2 | 1.59 | | 0.14 | | | 2.09 | | | 0.37 | | | 1.85 | 0.30 | 2.10 | | | 0.40 | 2.20 | 0.34 | 1.78 | | | | 0.40 | 3.86 | | 1.78 | 2.93 | | 0.84 |
| 10.17 | C3 | 1.11 | | 0.10 | | | 1.11 | | | 0.21 | | | 0.99 | 0.18 | 1.10 | | | 0.22 | 1.17 | 0.19 | 0.98 | | | | 0.22 | 2.08 | | 1.03 | 1.78 | | 0.62 |
| 10.32 | C4 | 4.65 | | 0.41 | | | 6.15 | | | 1.16 | | | 5.31 | 0.89 | 6.00 | | | 1.13 | 6.30 | 0.96 | 5.29 | | | | 1.18 | 11.38 | | 5.46 | 9.10 | | 2.86 |
| 10.47 | C5 | 1.68 | | 0.15 | | | 2.46 | | | 0.53 | | | 1.96 | 0.26 | 2.24 | | | 0.47 | 2.40 | 0.37 | 2.05 | | | | 0.48 | 4.77 | | 2.48 | 3.82 | | 1.30 |
| 10.7 | C6 | 1.85 | | 0.17 | | | 2.08 | | | 0.35 | | | 1.14 | 0.19 | 1.47 | | | 0.37 | 1.47 | 0.21 | 1.53 | | | | 0.41 | 2.72 | | 1.20 | 1.94 | | 0.59 |
| 11.25 | C7 | 3.20 | | 1.78 | | | 0.00 | | | 0.00 | | | 0.00 | 0.00 | 0.00 | | | 0.00 | 0.00 | 0.00 | 0.00 | | | | 0.00 | 0.00 | | 0.00 | 0.00 | | 0.00 |
| 14.7 | C9 | 1.88 | | 0.59 | | | | 0.00 | | 0.00 | | | 0.00 | 0.00 | 0.00 | | | 0.00 | 0.00 | 0.00 | 0.00 | | | 0.00 | | 0.00 | | 0.00 | 0.00 | | 0.00 |
| 18.09 | C10 | 1.67 | | 0.28 | | | | 0.00 | | 0.00 | | | 0.00 | 0.00 | 0.00 | | | 0.00 | 0.00 | 0.00 | 0.00 | | | 0.00 | | 0.00 | | 0.00 | 0.00 | | 0.00 |
| 24.04 | C11 | 3.07 | | 0.40 | | | | 0.00 | | 0.00 | | | 0.00 | 0.00 | 0.00 | | | 0.00 | 0.00 | 0.00 | 0.00 | | | 0.00 | | 0.00 | | 0.00 | 0.00 | | 0.00 |
| 29.63 | C15 | 1.95 | | 0.28 | | | | | 0.00 | | 0.00 | | 0.00 | 0.00 | 0.00 | | 0.00 | | 0.00 | 0.00 | | 0.00 | | 0.00 | | 0.00 | | 0.00 | 0.00 | | 0.00 |
| 31.14 | C16 | 0.90 | | 0.28 | | | | | 1.82 | | 1.09 | | 0.41 | 0.24 | 0.45 | | 0.21 | | 0.31 | 0.19 | | 0.10 | | 0.10 | | 0.91 | | 0.62 | 0.81 | | 0.38 |
| 31.25 | C17 | 0.86 | | 0.42 | | | | | 2.44 | | 1.42 | | 0.53 | 0.31 | 0.56 | | 0.25 | | 0.41 | 0.26 | | 0.13 | | 0.13 | | 1.07 | | 0.76 | 1.03 | | 0.49 |
| 31.58 | C18 | 15.52 | | 7.55 | | | | | 0.00 | | 0.00 | | 0.00 | 0.00 | 0.00 | | 0.00 | | 0.00 | 0.00 | | 0.00 | | 0.00 | | 0.00 | | 0.00 | 0.00 | | 0.00 |
| 33.65 | C19 | 1.24 | | 0.24 | | | | | 0.00 | | 0.00 | | 0.00 | 0.00 | 0.00 | | 0.00 | | 0.00 | 0.00 | | 0.00 | | 0.00 | | 0.00 | | 0.00 | 0.00 | | 0.00 |
| 33.31 | C20 | 3.74 | | 1.92 | | | | | 0.00 | | 0.00 | | 0.00 | 0.00 | 0.00 | | 0.00 | | 0.00 | 0.00 | | 0.00 | | 0.00 | | 0.00 | | 0.00 | 0.00 | | 0.00 |
|  |  |  | |  | | | | |  | |  | |  |  |  | |  | |  |  | |  | |  | |  | |  |  | |  |
|  | **Aldehydes** |  | |  | | | | |  | |  | |  |  |  | |  | |  |  | |  | |  | |  | |  |  | |  |
| 9.86 | hexanal | 1.24 | | 0.38 | | | | | 0.00 | | 0.00 | | 0.00 | 0.00 | 0.00 | | 0.00 | | 0.00 | 0.00 | | 0.00 | | 0.00 | | 0.74 | | 0.74 | 1.44 | | 1.24 |
| 13.22 | trans-2-hexenal | 3.81 | | 1.44 | | | | | 0.00 | | 0.00 | | 0.00 | 0.00 | 0.00 | | 0.00 | | 0.00 | 0.00 | | 0.00 | | 0.00 | | 8.13 | | 8.13 | 14.05 | | 3.81 |
| 16.41 | 6-methyl-5-hepten-2-one | traces | | | | | | | | | | | | | | | | | | | | | | | | | | | | | |
| 17.93 | nonanal | 2.26 | | 0.11 | | | | | 2.27 | | | 0.86 | 1.05 | 0.32 | | 1.73 | 0.31 | | 1.24 | 0.32 | | | 1.40 | 0.22 | | | 4.72 | 3.10 | | 5.60 | 2.26 |
| 21.17 | benzaldehyde | 35.96 | | 12.49 | | | | | 0.00 | | | 0.00 | 0.00 | 0.00 | | 0.00 | 0.00 | | 0.00 | 0.00 | | | 0.00 | 0.00 | | | 0.00 | 0.00 | | 0.13 | 35.96 |
| 24.15 | benzenacetaldehyde | 2.35 | | 0.41 | | | | | 0.00 | | | 0.00 | 0.00 | 0.00 | | 0.00 | 0.00 | | 0.00 | 0.00 | | | 0.00 | 0.00 | | | 0.00 | 0.00 | | 0.16 | 2.35 |
|  |  |  | |  | | | | |  | | |  |  |  | |  |  | |  |  | | |  |  | | |  |  | |  |  |
|  | **Nitrogen containing compounds** | | | | | | | | | | | |  |  | |  |  | |  |  | | |  |  | | |  |  | |  |  |
| 9.93 | 2-methylbutannitril | | 0.88 | | 0.09 | | | 0.00 | | 0.00 | | | 0.00 | 0.00 | | 0.00 | 0.00 | | 0.00 | 0.00 | | | 0.00 | 0.00 | | | 0.00 | 0.00 | | 0.00 | 0.00 |
| 10.81 | 3-methylbutannitril | | 2.04 | | 0.11 | | | 0.00 | | 0.00 | | | 0.00 | 0.00 | | 0.00 | 0.00 | | 0.00 | 0.00 | | | 0.00 | 0.00 | | | 0.00 | 0.00 | | 0.00 | 0.00 |
| 17.68 | (*E*)-isobutyraldoxime | | 1.30 | | 0.20 | | | 0.00 | | 0.00 | | | 0.00 | 0.00 | | 0.00 | 0.00 | | 0.00 | 0.00 | | | 0.00 | 0.00 | | | 0.00 | 0.00 | | 0.00 | 0.00 |
| 18.17 | (*Z*)-isobutyraldoxime | | 0.58 | | 0.12 | | | 0.00 | | 0.00 | | | 0.00 | 0.00 | | 0.00 | 0.00 | | 0.00 | 0.00 | | | 0.00 | 0.00 | | | 0.00 | 0.00 | | 0.00 | 0.00 |
| 20.40 | (*E*)-2-methyl-butyraldoxime | | 22.61 | | 2.63 | | | 0.00 | | 0.00 | | | 0.00 | 0.00 | | 0.00 | 0.00 | | 0.00 | 0.00 | | | 0.00 | 0.00 | | | 0.00 | 0.00 | | 0.00 | 0.00 |
| 20.55 | (*E*)-3-methyl-butyraldoxime | | 19.83 | | 2.77 | | | 0.00 | | 0.00 | | | 0.00 | 0.00 | | 0.00 | 0.00 | | 0.00 | 0.00 | | | 0.00 | 0.00 | | | 0.00 | 0.00 | | 0.00 | 0.00 |
| 20.86 | (*Z*)-2-methyl-butyraldoxime | | 7.77 | | 1.03 | | | 0.00 | | 0.00 | | | 0.00 | 0.00 | | 0.00 | 0.00 | | 0.00 | 0.00 | | | 0.00 | 0.00 | | | 0.00 | 0.00 | | 0.00 | 0.00 |
| 21.48 | (*Z*)-3-methyl-butyraldoxime | | 12.91 | | 1.79 | | | 0.00 | | 0.00 | | | 0.00 | 0.00 | | 0.00 | 0.00 | | 0.00 | 0.00 | | | 0.00 | 0.00 | | | 0.00 | 0.00 | | 0.00 | 0.00 |
| 30.30 | benzyl cyanide | | 55.29 | | 11.24 | | | 0.00 | | 0.00 | | | 0.00 | 0.00 | | 0.00 | 0.00 | | 0.00 | 0.00 | | | 0.00 | 0.00 | | | 0.00 | 0.00 | | 0.00 | 0.00 |
| 34.06 | phenylnitroethane | | 16.60 | | 2.41 | | | 0.00 | | 0.00 | | | 0.00 | 0.00 | | 0.00 | 0.00 | | 0.00 | 0.00 | | | 0.00 | 0.00 | | | 0.00 | 0.00 | | 0.00 | 0.00 |
| 37.16 | (*E*)-phenylacetaldoxime | | 0.38 | | 0.07 | | | 0.00 | | 0.00 | | | 0.00 | 0.00 | | 0.00 | 0.00 | | 0.00 | 0.00 | | | 0.00 | 0.00 | | | 0.00 | 0.00 | | 0.00 | 0.00 |
| 37.88 | (*Z*)-phenylacetaldoxime | | 0.66 | | 0.11 | | | 0.00 | | 0.00 | | | 0.00 | 0.00 | | 0.00 | 0.00 | | 0.00 | 0.00 | | | 0.00 | 0.00 | | | 0.00 | 0.00 | | 0.00 | 0.00 |
| 39.90 | indole | | 56.73 | | 9.11 | | | 0.85 | | 0.61 | | | 0.41 | 0.41 | | 0.00 | 0.00 | | 0.00 | 0.00 | | | 0.00 | 0.00 | | | 0.25 | 0.25 | | 0.00 | 0.00 |
|  |  | |  | |  | | |  | |  | | |  |  | |  |  | |  |  | | |  |  | | |  |  | |  |  |
|  | **Monoterpenes** | |  | |  | | |  | |  | | |  |  | |  |  | |  |  | | |  |  | | |  |  | |  |  |
| 8.73 | α-pinene | | traces | | | | | 0.00 | | 0.00 | | | 0.00 | 0.00 | | 0.00 | 0.00 | | 0.00 | 0.00 | | | 0.00 | 0.00 | | | 0.00 | 0.00 | | 0.00 | 0.00 |
| 11.88 | myrcene | | 1.58 | | 0.23 | | | 0.00 | | 0.00 | | | 0.00 | 0.00 | | 0.00 | 0.00 | | 0.00 | 0.00 | | | 0.00 | 0.00 | | | 0.00 | 0.00 | | 0.00 | 0.00 |
| 12.78 | limonene | | 0.43 | | 0.27 | | | 0.00 | | 0.00 | | | 0.00 | 0.00 | | 0.00 | 0.00 | | 0.00 | 0.00 | | | 0.00 | 0.00 | | | 0.00 | 0.00 | | 0.55 | 0.55 |
| 13.13 | 1,8-cineole | | traces | | | | | 0.00 | | 0.00 | | | 0.00 | 0.00 | | 0.00 | 0.00 | | 0.00 | 0.00 | | | 0.00 | 0.00 | | | 0.00 | 0.00 | | 0.00 | 0.00 |
| 13.70 | (*Z*)-β-ocimene | | 16.23 | | 1.80 | | | 0.25 | | 0.25 | | | 0.00 | 0.00 | | 0.00 | 0.00 | | 0.00 | 0.00 | | | 0.00 | 0.00 | | | 0.00 | 0.00 | | 0.00 | 0.00 |
| 14.16 | (*E*)- β-ocimene | | 389.0 | | 54.86 | | | 2.84 | | 0.71 | | | 1.09 | 0.57 | | 2.25 | 1.46 | | 0.44 | 0.28 | | | 0.92 | 0.08 | | | 0.95 | 0.56 | | 1.07 | 0.68 |
| 17.35 | alloocimene | | 1.00 | | 0.12 | | | 0.00 | | 0.00 | | | 0.00 | 0.00 | | 0.00 | 0.00 | | 0.00 | 0.00 | | | 0.00 | 0.00 | | | 0.00 | 0.00 | | 0.00 | 0.00 |
| 21.75 | linalool | | 5.55 | | 3.17 | | | 6.84 | | 5.46 | | | 0.33 | 0.33 | | 0.97 | 0.44 | | 0.70 | 0.48 | | | 0.43 | 0.26 | | | 0.50 | 0.21 | | 0.95 | 0.55 |
| 20.30 | (*E*)-epoxy-ocimene | | 2.19 | | 0.29 | | | 0.00 | | 0.00 | | | 0.00 | 0.00 | | 0.00 | 0.00 | | 0.00 | 0.00 | | | 0.00 | 0.00 | | | 0.00 | 0.00 | | 0.00 | 0.00 |
| 26.87 | citronellol | | traces | | | | | 0.00 | | 0.00 | | | 0.00 | 0.00 | | 0.00 | 0.00 | | 0.00 | 0.00 | | | 0.00 | 0.00 | | | 0.00 | 0.00 | | 0.00 | 0.00 |
|  |  | |  | | |  | |  | |  | | |  |  | |  |  | |  |  | | |  |  | | |  |  | |  |  |
|  | **Homoterpenes** | |  | | |  | |  | |  | | |  |  | |  |  | |  |  | | |  |  | | |  |  | |  |  |
| 15.66 | DMNT | | 3.10 | | | 1.12 | | 3.57 | | 3.23 | | | 0.41 | 0.41 | | 0.68 | 0.42 | | 0.15 | 0.15 | | | 0.13 | 0.13 | | | 1.10 | 0.78 | | 0.69 | 0.42 |
| 27.86 | TMTT | | 3.13 | | | 1.44 | | 3.14 | | 3.14 | | | 0.00 | 0.00 | | 0.00 | 0.00 | | 0.00 | 0.00 | | | 0.00 | 0.00 | | | 0.00 | 0.00 | | 0.62 | 0.42 |
|  |  | |  | | |  | |  | |  | | |  |  | |  |  | |  |  | | |  |  | | |  |  | |  |  |
|  | **Alcohols** | |  | | |  | |  | |  | | |  |  | |  |  | |  |  | | |  |  | | |  |  | |  |  |
| 12.90 | 2/3-methylbutanol | | traces | | | | | 0.00 | | 0.00 | | | 0.00 | 0.00 | | 0.00 | 0.00 | | 0.00 | 0.00 | | | 0.00 | 0.00 | | | 0.00 | 0.00 | | 0.00 | 0.00 |
| 16.82 | 1-hexanol | | 14.40 | | 3.18 | | | 0.00 | | 0.00 | | | 0.00 | 0.00 | | 0.26 | 0.26 | | 0.00 | 0.00 | | | 0.00 | 0.00 | | | 11.81 | 11.81 | | 10.47 | 10.23 |
| 17.62 | (*Z*)-3-hexenol | | 20.19 | | 5.18 | | | 4.11 | | 2.12 | | | 1.02 | 0.72 | | 16.51 | 10.85 | | 3.51 | 1.78 | | | 2.21 | 0.99 | | | 224.5 | 222.5 | | 258.9 | 257.5 |
| 18.19 | (*Z*)-2-hexenol | | 1.07 | | 0.33 | | | 0.00 | | 0.00 | | | 0.00 | 0.00 | | 0.00 | 0.00 | | 0.00 | 0.00 | | | 0.00 | 0.00 | | | 3.67 | 3.67 | | 4.80 | 4.15 |
| 22.04 | 1-octanol | | 0.57 | | 0.15 | | | 0.85 | | 0.47 | | | 0.00 | 0.00 | | 0.00 | 0.00 | | 0.16 | 0.16 | | | 0.25 | 0.16 | | | 7.99 | 7.71 | | 7.44 | 6.51 |
| 24.50 | nonanol | | 1.27 | | 0.21 | | | 1.68 | | 0.57 | | | 0.17 | 0.17 | | 0.88 | 0.26 | | 0.72 | 0.31 | | | 0.80 | 0.31 | | | 1.37 | 0.41 | | 3.20 | 0.94 |
| 29.30 | benzyl alcohol | | 8.58 | | 1.00 | | | 0.83 | | 0.53 | | | 0.00 | 0.00 | | 0.35 | 0.22 | | 0.09 | 0.09 | | | 0.23 | 0.13 | | | 0.24 | 0.15 | | 0.27 | 0.27 |
| 29.98 | phenylethyl alcohol | | 48.20 | | 9.20 | | | 0.44 | | 0.23 | | | 0.17 | 0.17 | | 0.00 | 0.00 | | 0.25 | 0.25 | | | 0.00 | 0.00 | | | 1.10 | 0.94 | | 0.00 | 0.00 |
| 31.14 | 1.2-(*E*)-cyclohexandiol | | 5.74 | | 2.06 | | | 0.00 | | 0.00 | | | 0.00 | 0.00 | | 0.00 | 0.00 | | 0.00 | 0.00 | | | 0.00 | 0.00 | | | 0.00 | 0.00 | | 0.00 | 0.00 |
|  |  | |  | |  | | |  | |  | | |  |  | |  |  | |  |  | | |  |  | | |  |  | |  |  |
|  | **Esters** | |  | |  | | |  | |  | | |  |  | |  |  | |  |  | | |  |  | | |  |  | |  |  |
| 15.87 | (*Z*)-3-hexenyl acetate | | 10.03 | | 1.63 | | | 3.24 | | 1.72 | | | 1.11 | 0.71 | | 10.35 | 7.14 | | 2.39 | 1.24 | | | 1.92 | 1.08 | | | 39.05 | 38.46 | | 22.21 | 22.21 |
| 19.67 | (*Z*)-3-hexenyl butyrate | | 2.20 | | 0.35 | | | 0.00 | | 0.00 | | | 0.00 | 0.00 | | 0.00 | 0.00 | | 0.00 | 0.00 | | | 0.00 | 0.00 | | | 0.00 | 0.00 | | 0.00 | 0.00 |
| 20.05 | (*Z*)-3-hexenyl-2-methyl butyrate | | 3.03 | | 0.63 | | | 0.24 | | 0.24 | | | 0.00 | 0.00 | | 0.20 | 0.20 | | 0.00 | 0.00 | | | 0.00 | 0.00 | | | 0.78 | 0.78 | | 1.60 | 1.60 |
| 27.16 | methyl salicylate | | 0.69 | | 0.30 | | | 0.24 | | 0.24 | | | 0.00 | 0.00 | | 0.08 | 0.08 | | 0.00 | 0.00 | | | 0.07 | 0.07 | | | 0.00 | 0.00 | | 0.55 | 0.55 |
| 27.97 | phenylethyl acetate | | 0.79 | | 0.57 | | | 0.00 | | 0.00 | | | 0.00 | 0.00 | | 0.00 | 0.00 | | 0.00 | 0.00 | | | 0.00 | 0.00 | | | 0.00 | 0.00 | | 0.00 | 0.00 |
| 34.28 | (*Z*)-3-hexenyl benzoate | | 11.97 | | 4.88 | | | 1.01 | | 1.01 | | | 0.00 | 0.00 | | 0.34 | 0.34 | | 0.12 | 0.12 | | | 0.00 | 0.00 | | | 0.00 | 0.00 | | 0.44 | 0.44 |
|  |  | |  | |  | | |  | |  | | |  |  | |  |  | |  |  | | |  |  | | |  |  | |  |  |
|  | **Sesquiterpenes** | |  | |  | | |  | |  | | |  |  | |  |  | |  |  | | |  |  | | |  |  | |  |  |
| 19.76 | α-cubebene | | 0.79 | | 0.32 | | | 0.00 | | 0.00 | | | 0.00 | 0.00 | | 0.00 | 0.00 | | 0.00 | 0.00 | | | 0.00 | 0.00 | | | 0.00 | 0.00 | | 0.00 | 0.79 |
| 20.63 | α-copaene | | traces | | | | | 0.00 | | 0.00 | | | 0.00 | 0.00 | | 0.00 | 0.00 | | 0.00 | 0.00 | | | 0.00 | 0.00 | | | 0.00 | 0.00 | | 0.00 | 0.00 |
| 23.20 | (*E*)-β-caryophyllene | | 25.72 | | 10.96 | | | 23.80 | | 8.61 | | | 5.49 | 0.90 | | 8.16 | 2.58 | | 10.60 | 5.10 | | | 10.36 | 3.95 | | | 20.03 | 11.20 | | 7.40 | 2.48 |
| 24.70 | (*E*)- β-farnesene | | 1.03 | | 0.31 | | | 0.00 | | 0.00 | | | 0.00 | 0.00 | | 0.00 | 0.00 | | 0.00 | 0.00 | | | 0.00 | 0.00 | | | 0.00 | 0.00 | | 0.00 | 0.00 |
| 24.90 | α-humulene | | 1.55 | | 0.67 | | | 0.89 | | 0.59 | | | 0.00 | 0.00 | | 0.08 | 0.08 | | 0.32 | 0.32 | | | 0.45 | 0.26 | | | 0.26 | 0.26 | | 0.30 | 0.20 |
| 25.80 | germacrene D | | 19.43 | | 4.95 | | | 0.00 | | 0.00 | | | 0.00 | 0.00 | | 0.00 | 0.00 | | 0.00 | 0.00 | | | 0.00 | 0.00 | | | 0.00 | 0.00 | | 0.00 | 0.00 |
| 26.03 | (*Z*.*E*)-α-farnesene | | 27.24 | | 5.82 | | | 2.61 | | 1.70 | | | 0.21 | 0.21 | | 0.66 | 0.45 | | 0.47 | 0.29 | | | 0.86 | 0.59 | | | 2.40 | 1.46 | | 2.58 | 1.47 |
| 26.57 | (*E*.*E*)-α-farnesene | | 796.8 | | 100.4 | | | 84.73 | | 43.63 | | | 18.07 | 10.02 | | 42.22 | 16.95 | | 25.04 | 8.83 | | | 24.97 | 12.78 | | | 37.69 | 13.64 | | 46.02 | 28.01 |
| 26.80 | δ-cadinene | | 1.82 | | 0.56 | | | 1.17 | | 1.17 | | | 0.00 | 0.00 | | 0.00 | 0.00 | | 0.00 | 0.00 | | | 0.00 | 0.00 | | | 0.00 | 0.00 | | 0.46 | 0.46 |
| 32.56 | nerolidol | | 0.42 | | 0.19 | | | 0.76 | | 0.76 | | | 0.00 | 0.00 | | 0.00 | 0.00 | | 0.00 | 0.00 | | | 0.00 | 0.00 | | | 0.00 | 0.00 | | 0.44 | 0.44 |

**(C)**

| **RT** |  | **ctr-3** | | SE | | | **ctr-4** | | SE | | | **ctr-5** | SE | **ctr-6** | | | SE | **ctr-7** | SE | **ctr-8** | | | | SE | **ctr-9** | | SE | **ctr-10** | | SE |
| --- | --- | --- | --- | --- | --- | --- | --- | --- | --- | --- | --- | --- | --- | --- | --- | --- | --- | --- | --- | --- | --- | --- | --- | --- | --- | --- | --- | --- | --- | --- |
|  | **Unidentified** |  | |  | | |  | |  | | |  |  |  | | |  |  |  |  | | | |  |  | |  |  | |  |
| 9.20 | C1 | 4.15 | | 1.20 | | | 3.85 | | 1.00 | | | 4.31 | 1.29 | 5.57 | | | 1.08 | 4.84 | 1.47 | 5.65 | | | | 1.88 | 5.31 | | 1.76 | 9.23 | | 2.60 |
| 9.30 | C2 | 1.71 | | 0.49 | | | 1.60 | | 0.42 | | | 1.77 | 0.54 | 2.26 | | | 0.43 | 2.12 | 0.67 | 2.11 | | | | 0.89 | 2.33 | | 0.79 | 3.84 | | 1.11 |
| 10.17 | C3 | 0.93 | | 0.27 | | | 0.86 | | 0.22 | | | 0.94 | 0.29 | 1.22 | | | 0.24 | 0.98 | 0.28 | 1.26 | | | | 0.42 | 1.10 | | 0.35 | 2.10 | | 0.60 |
| 10.32 | C4 | 4.96 | | 1.44 | | | 4.66 | | 1.20 | | | 5.16 | 1.59 | 6.61 | | | 1.25 | 5.57 | 1.64 | 6.80 | | | | 2.28 | 6.15 | | 2.01 | 11.32 | | 3.27 |
| 10.47 | C5 | 1.86 | | 0.53 | | | 1.79 | | 0.46 | | | 2.07 | 0.67 | 2.43 | | | 0.45 | 2.13 | 0.62 | 2.59 | | | | 0.89 | 2.03 | | 0.66 | 4.62 | | 1.42 |
| 10.7 | C6 | 1.41 | | 0.42 | | | 1.28 | | 0.33 | | | 1.32 | 0.38 | 1.95 | | | 0.53 | 1.47 | 0.46 | 2.01 | | | | 0.81 | 1.74 | | 0.60 | 3.04 | | 1.06 |
| 11.25 | C7 | 0.00 | | 0.00 | | | 0.00 | | 0.00 | | | 0.00 | 0.00 | 0.00 | | | 0.00 | 0.00 | 0.00 | 0.00 | | | | 0.00 | 0.00 | | 0.00 | 0.00 | | 0.00 |
| 14.7 | C9 | 0.00 | | 0.00 | | | | 0.18 | 0.18 | | | 0.00 | 0.00 | 0.00 | | | 0.00 | 0.00 | 0.00 | 0.00 | | | 0.00 | | 0.00 | | 0.00 | 0.00 | | 0.00 |
| 18.09 | C10 | 0.00 | | 0.00 | | | | 0.15 | 0.15 | | | 0.00 | 0.00 | 0.00 | | | 0.00 | 0.00 | 0.00 | 0.00 | | | 0.00 | | 0.15 | | 0.15 | 0.00 | | 0.00 |
| 24.04 | C11 | 0.00 | | 0.00 | | | | 0.00 | 0.00 | | | 0.00 | 0.00 | 0.00 | | | 0.00 | 0.00 | 0.00 | 0.00 | | | 0.00 | | 0.00 | | 0.00 | 0.00 | | 0.00 |
| 29.63 | C15 | 0.00 | | 0.00 | | | | 0.00 | | 0.00 | | 0.00 | 0.00 | 0.00 | | 0.00 | | 0.00 | 0.00 | | 0.00 | | 0.00 | | 0.16 | | 0.16 | 0.00 | | 0.00 |
| 31.14 | C16 | 1.03 | | 0.52 | | | | 2.26 | | 1.02 | | 1.37 | 0.65 | 1.29 | | 1.08 | | 0.20 | 0.20 | | 0.64 | | 0.39 | | 0.95 | | 0.55 | 0.57 | | 0.47 |
| 31.25 | C17 | 1.48 | | 0.66 | | | | 1.25 | | 0.41 | | 1.92 | 1.00 | 1.91 | | 1.38 | | 0.75 | 0.35 | | 1.47 | | 0.46 | | 0.98 | | 0.69 | 0.62 | | 0.52 |
| 31.58 | C18 | 0.00 | | 0.00 | | | | 0.00 | | 0.00 | | 0.00 | 0.00 | 0.00 | | 0.00 | | 0.00 | 0.00 | | 0.00 | | 0.00 | | 0.00 | | 0.00 | 0.00 | | 0.00 |
| 33.65 | C19 | 0.00 | | 0.00 | | | | 0.40 | | 0.40 | | 0.00 | 0.00 | 0.00 | | 0.00 | | 0.00 | 0.00 | | 0.00 | | 0.00 | | 0.00 | | 0.00 | 0.00 | | 0.00 |
| 33.31 | C20 | 0.00 | | 0.00 | | | | 0.00 | | 0.00 | | 0.00 | 0.00 | 0.00 | | 0.00 | | 0.00 | 0.00 | | 0.00 | | 0.00 | | 0.00 | | 0.00 | 0.00 | | 0.00 |
|  |  |  | |  | | | |  | |  | |  |  |  | |  | |  |  | |  | |  | |  | |  |  | |  |
|  | **Aldehydes** |  | |  | | | |  | |  | |  |  |  | |  | |  |  | |  | |  | |  | |  |  | |  |
| 9.86 | hexanal | 0.00 | | 0.00 | | | | 0.00 | | 0.00 | | 0.00 | 0.00 | 0.00 | | 0.00 | | 0.00 | 0.00 | | 0.00 | | 0.00 | | 0.00 | | 0.00 | 0.57 | | 0.57 |
| 13.22 | trans-2-hexenal | 0.00 | | 0.00 | | | | 0.00 | | 0.00 | | 0.00 | 0.00 | 0.00 | | 0.00 | | 0.00 | 0.00 | | 0.00 | | 0.00 | | 0.00 | | 0.00 | 0.00 | | 0.00 |
| 16.41 | 6-methyl-5-hepten-2-one | traces | | | | | | | | | | | | | | | | | | | | | | | | | | | | |
| 17.93 | nonanal | 1.53 | | 0.29 | | | | 2.68 | | | 1.13 | 2.52 | 0.72 | | 2.88 | 0.68 | | 5.17 | 3.09 | | | 2.68 | 0.62 | | | 7.09 | 3.91 | | 4.87 | 1.77 |
| 21.17 | benzaldehyde | 0.25 | | 0.16 | | | | 0.14 | | | 0.14 | 0.39 | 0.39 | | 0.12 | 0.12 | | 0.97 | 0.97 | | | 0.00 | 0.00 | | | 0.08 | 0.08 | | 0.43 | 0.43 |
| 24.15 | benzenacetaldehyde | 0.00 | | 0.00 | | | | 0.23 | | | 0.23 | 0.00 | 0.00 | | 0.00 | 0.00 | | 0.00 | 0.00 | | | 0.00 | 0.00 | | | 0.12 | 0.12 | | 0.37 | 0.37 |
|  |  |  | |  | | | |  | | |  |  |  | |  |  | |  |  | | |  |  | | |  |  | |  |  |
|  | **Nitrogen containing compounds** | | | | | | | | | | |  |  | |  |  | |  |  | | |  |  | | |  |  | |  |  |
| 9.93 | 2-methylbutyronitrile | | 0.00 | | 0.00 | | | 0.00 | 0.00 | | | 0.00 | 0.00 | | 0.00 | 0.00 | | 0.00 | 0.00 | | | 0.00 | 0.00 | | | 0.00 | 0.00 | | 0.00 | 0.00 |
| 10.81 | 3-methylbutyronitrile | | 0.00 | | 0.00 | | | 0.00 | 0.00 | | | 0.00 | 0.00 | | 0.00 | 0.00 | | 0.00 | 0.00 | | | 0.00 | 0.00 | | | 0.00 | 0.00 | | 0.00 | 0.00 |
| 17.68 | (*E*)-isobutyraldoxime | | 0.00 | | 0.00 | | | 0.00 | 0.00 | | | 0.00 | 0.00 | | 0.00 | 0.00 | | 0.00 | 0.00 | | | 0.00 | 0.00 | | | 0.00 | 0.00 | | 0.00 | 0.00 |
| 18.17 | (*Z*)-isobutyraldoxime | | 0.00 | | 0.00 | | | 0.00 | 0.00 | | | 0.00 | 0.00 | | 0.00 | 0.00 | | 0.00 | 0.00 | | | 0.00 | 0.00 | | | 0.00 | 0.00 | | 0.00 | 0.00 |
| 20.40 | (*E*)-2-methyl-butyraldoxime | | 0.00 | | 0.00 | | | 0.00 | 0.00 | | | 0.00 | 0.00 | | 0.00 | 0.00 | | 0.00 | 0.00 | | | 0.00 | 0.00 | | | 0.00 | 0.00 | | 0.00 | 0.00 |
| 20.55 | (*E*)-3-methyl-butyraldoxime | | 0.00 | | 0.00 | | | 0.00 | 0.00 | | | 0.00 | 0.00 | | 0.00 | 0.00 | | 0.00 | 0.00 | | | 0.00 | 0.00 | | | 0.00 | 0.00 | | 0.00 | 0.00 |
| 20.86 | (*Z*)-2-methyl-butyraldoxime | | 0.00 | | 0.00 | | | 0.00 | 0.00 | | | 0.00 | 0.00 | | 0.00 | 0.00 | | 0.00 | 0.00 | | | 0.00 | 0.00 | | | 0.00 | 0.00 | | 0.00 | 0.00 |
| 21.48 | (*Z*)-3-methyl-butyraldoxime | | 0.00 | | 0.00 | | | 0.00 | 0.00 | | | 0.00 | 0.00 | | 0.00 | 0.00 | | 0.00 | 0.00 | | | 0.00 | 0.00 | | | 0.00 | 0.00 | | 0.00 | 0.00 |
| 30.30 | benzyl cyanide | | 0.00 | | 0.00 | | | 1.38 | 0.86 | | | 0.37 | 0.37 | | 0.10 | 0.10 | | 0.35 | 0.35 | | | 0.00 | 0.00 | | | 0.15 | 0.15 | | 0.00 | 0.00 |
| 34.06 | phenylnitroethane | | 0.00 | | 0.00 | | | 0.16 | 0.16 | | | 0.00 | 0.00 | | 0.00 | 0.00 | | 0.00 | 0.00 | | | 0.00 | 0.00 | | | 0.00 | 0.00 | | 0.00 | 0.00 |
| 37.16 | (*E*)-phenylacetaldoxime | | 0.00 | | 0.00 | | | 0.00 | 0.00 | | | 0.00 | 0.00 | | 0.00 | 0.00 | | 0.00 | 0.00 | | | 0.00 | 0.00 | | | 0.00 | 0.00 | | 0.00 | 0.00 |
| 37.88 | (*Z*)-phenylacetaldoxime | | 0.00 | | 0.00 | | | 0.00 | 0.00 | | | 0.00 | 0.00 | | 0.00 | 0.00 | | 0.00 | 0.00 | | | 0.00 | 0.00 | | | 0.00 | 0.00 | | 0.00 | 0.00 |
| 39.90 | indole | | 0.69 | | 0.48 | | | 6.76 | 6.26 | | | 1.57 | 1.39 | | 0.00 | 0.00 | | 0.00 | 0.00 | | | 0.00 | 0.00 | | | 0.08 | 0.08 | | 0.00 | 0.00 |
|  |  | |  | |  | | |  |  | | |  |  | |  |  | |  |  | | |  |  | | |  |  | |  |  |
|  | **Monoterpenes** | |  | |  | | |  |  | | |  |  | |  |  | |  |  | | |  |  | | |  |  | |  |  |
| 8.73 | α-pinene | | 0.00 | | 0.00 | | | 0.00 | 0.00 | | | 0.00 | 0.00 | | 0.00 | 0.00 | | 0.00 | 0.00 | | | 0.00 | 0.00 | | | 0.00 | 0.00 | | 0.00 | 0.00 |
| 11.88 | myrcene | | 0.10 | | 0.10 | | | 0.31 | 0.19 | | | 0.00 | 0.00 | | 0.00 | 0.00 | | 0.00 | 0.00 | | | 0.00 | 0.00 | | | 0.00 | 0.00 | | 0.00 | 0.00 |
| 12.78 | limonene | | 0.00 | | 0.00 | | | 0.00 | 0.00 | | | 0.00 | 0.00 | | 0.00 | 0.00 | | 0.00 | 0.00 | | | 0.00 | 0.00 | | | 0.00 | 0.00 | | 0.57 | 0.57 |
| 13.13 | 1,8-cineole | | 0.00 | | 0.00 | | | 0.00 | 0.00 | | | 0.00 | 0.00 | | 0.00 | 0.00 | | 0.00 | 0.00 | | | 0.00 | 0.00 | | | 0.00 | 0.00 | | 0.00 | 0.00 |
| 13.70 | (*Z*)-β-ocimene | | 0.00 | | 0.00 | | | 1.19 | 0.77 | | | 0.36 | 0.36 | | 0.15 | 0.15 | | 0.00 | 0.00 | | | 0.00 | 0.00 | | | 0.08 | 0.08 | | 0.00 | 0.00 |
| 14.16 | (*E*)- β-ocimene | | 2.59 | | 0.42 | | | 22.04 | 14.37 | | | 7.50 | 5.83 | | 2.76 | 1.30 | | 3.19 | 1.70 | | | 0.24 | 0.24 | | | 1.74 | 1.05 | | 1.02 | 0.46 |
| 17.35 | alloocimene | | 0.00 | | 0.00 | | | 0.00 | 0.00 | | | 0.00 | 0.00 | | 0.00 | 0.00 | | 0.00 | 0.00 | | | 0.00 | 0.00 | | | 0.00 | 0.00 | | 0.00 | 0.00 |
| 21.75 | linalool | | 2.00 | | 0.95 | | | 2.51 | 1.42 | | | 1.79 | 0.65 | | 2.09 | 0.44 | | 1.44 | 0.73 | | | 0.16 | 0.16 | | | 0.88 | 0.68 | | 0.00 | 0.00 |
| 20.30 | (*E*)-epoxy-ocimene | | 0.00 | | 0.00 | | | 0.00 | 0.00 | | | 0.00 | 0.00 | | 0.00 | 0.00 | | 0.00 | 0.00 | | | 0.00 | 0.00 | | | 0.00 | 0.00 | | 0.00 | 0.00 |
| 26.87 | citronellol | | 0.00 | | 0.00 | | | 0.00 | 0.00 | | | 0.00 | 0.00 | | 0.00 | 0.00 | | 0.00 | 0.00 | | | 0.00 | 0.00 | | | 0.00 | 0.00 | | 0.00 | 0.00 |
|  |  | |  | | |  | |  |  | | |  |  | |  |  | |  |  | | |  |  | | |  |  | |  |  |
|  | **Homoterpenes** | |  | | |  | |  |  | | |  |  | |  |  | |  |  | | |  |  | | |  |  | |  |  |
| 15.66 | DMNT | | 0.37 | | | 0.23 | | 0.32 | 0.32 | | | 1.01 | 0.62 | | 0.23 | 0.23 | | 0.51 | 0.36 | | | 0.00 | 0.00 | | | 0.21 | 0.21 | | 0.00 | 0.00 |
| 27.86 | TMTT | | 0.00 | | | 0.00 | | 0.51 | 0.51 | | | 0.27 | 0.27 | | 0.00 | 0.00 | | 0.00 | 0.00 | | | 0.00 | 0.00 | | | 0.21 | 0.21 | | 0.00 | 0.00 |
|  |  | |  | | |  | |  |  | | |  |  | |  |  | |  |  | | |  |  | | |  |  | |  |  |
|  | **Alcohols** | |  | | |  | |  |  | | |  |  | |  |  | |  |  | | |  |  | | |  |  | |  |  |
| 12.90 | 2/3-methylbutanol | | 0.00 | | | 0.00 | | 0.00 | 0.00 | | | 0.00 | 0.00 | | 0.00 | 0.00 | | 0.00 | 0.00 | | | 0.00 | 0.00 | | | 0.00 | 0.00 | | 0.00 | 0.00 |
| 16.82 | 1-hexanol | | 0.00 | | 0.00 | | | 1.74 | 1.74 | | | 0.00 | 0.00 | | 0.59 | 0.43 | | 0.00 | 0.00 | | | 0.00 | 0.00 | | | 0.00 | 0.00 | | 0.63 | 0.46 |
| 17.62 | (*Z*)-3-hexenol | | 3.10 | | 0.83 | | | 1.27 | 0.48 | | | 6.45 | 3.78 | | 13.00 | 10.62 | | 5.27 | 2.71 | | | 14.68 | 8.99 | | | 21.90 | 12.38 | | 11.98 | 5.03 |
| 18.19 | (*Z*)-2-hexenol | | 0.00 | | 0.00 | | | 0.38 | 0.38 | | | 0.00 | 0.00 | | 0.00 | 0.00 | | 0.00 | 0.00 | | | 0.00 | 0.00 | | | 0.00 | 0.00 | | 1.02 | 1.02 |
| 22.04 | 1-octanol | | 0.16 | | 0.16 | | | 0.54 | 0.33 | | | 0.53 | 0.23 | | 0.86 | 0.64 | | 1.00 | 0.58 | | | 0.19 | 0.19 | | | 0.32 | 0.20 | | 0.55 | 0.41 |
| 24.50 | nonanol | | 0.54 | | 0.23 | | | 0.81 | 0.51 | | | 0.80 | 0.36 | | 1.94 | 0.58 | | 0.69 | 0.50 | | | 0.85 | 0.36 | | | 1.23 | 0.52 | | 2.38 | 0.36 |
| 29.30 | benzyl alcohol | | 1.50 | | 0.46 | | | 7.70 | 6.60 | | | 0.39 | 0.24 | | 0.37 | 0.37 | | 0.47 | 0.32 | | | 0.14 | 0.14 | | | 0.44 | 0.27 | | 0.11 | 0.11 |
| 29.98 | phenylethyl alcohol | | 1.00 | | 0.44 | | | 1.49 | 1.35 | | | 0.21 | 0.21 | | 0.17 | 0.17 | | 0.28 | 0.28 | | | 0.00 | 0.00 | | | 0.19 | 0.19 | | 0.00 | 0.00 |
| 31.14 | 1.2-(*E*)-cyclohexandiol | | 0.00 | | 0.00 | | | 0.00 | 0.00 | | | 0.00 | 0.00 | | 0.00 | 0.00 | | 0.00 | 0.00 | | | 0.00 | 0.00 | | | 0.00 | 0.00 | | 0.00 | 0.00 |
|  |  | |  | |  | | |  |  | | |  |  | |  |  | |  |  | | |  |  | | |  |  | |  |  |
|  | **Esters** | |  | |  | | |  |  | | |  |  | |  |  | |  |  | | |  |  | | |  |  | |  |  |
| 15.87 | (*Z*)-3-hexenyl acetate | | 1.28 | | 0.57 | | | 0.79 | 0.34 | | | 5.76 | 2.13 | | 9.66 | 7.13 | | 2.81 | 0.84 | | | 4.61 | 2.54 | | | 7.14 | 4.43 | | 3.14 | 1.22 |
| 19.67 | (*Z*)-3-hexenyl butyrate | | 0.00 | | 0.00 | | | 0.00 | 0.00 | | | 0.00 | 0.00 | | 0.00 | 0.00 | | 0.00 | 0.00 | | | 0.00 | 0.00 | | | 0.00 | 0.00 | | 0.00 | 0.00 |
| 20.05 | (*Z*)-3-hexenyl-2-methyl butyrate | | 0.00 | | 0.00 | | | 0.30 | 0.30 | | | 0.00 | 0.00 | | 0.00 | 0.00 | | 0.27 | 0.27 | | | 0.00 | 0.00 | | | 0.26 | 0.26 | | 0.00 | 0.00 |
| 27.16 | methyl salicylate | | 0.00 | | 0.00 | | | 0.00 | 0.00 | | | 0.00 | 0.00 | | 0.00 | 0.00 | | 0.00 | 0.00 | | | 0.00 | 0.00 | | | 0.00 | 0.00 | | 0.00 | 0.00 |
| 27.97 | phenylethyl acetate | | 0.00 | | 0.00 | | | 0.00 | 0.00 | | | 0.00 | 0.00 | | 0.00 | 0.00 | | 0.00 | 0.00 | | | 0.00 | 0.00 | | | 0.00 | 0.00 | | 0.00 | 0.00 |
| 34.28 | (*Z*)-3-hexenyl benzoate | | 0.98 | | 0.42 | | | 2.07 | 1.93 | | | 0.52 | 0.52 | | 0.34 | 0.34 | | 0.10 | 0.10 | | | 0.00 | 0.00 | | | 0.00 | 0.00 | | 0.00 | 0.00 |
|  |  | |  | |  | | |  |  | | |  |  | |  |  | |  |  | | |  |  | | |  |  | |  |  |
|  | **Sesquiterpenes** | |  | |  | | |  |  | | |  |  | |  |  | |  |  | | |  |  | | |  |  | |  |  |
| 19.76 | α-cubebene | | 0.00 | | 0.00 | | | 0.00 | 0.00 | | | 0.00 | 0.00 | | 0.00 | 0.00 | | 0.00 | 0.00 | | | 0.00 | 0.00 | | | 0.00 | 0.00 | | 0.33 | 0.33 |
| 20.63 | α-copaene | | 0.00 | | 0.00 | | | 0.00 | 0.00 | | | 0.00 | 0.00 | | 0.00 | 0.00 | | 0.00 | 0.00 | | | 0.00 | 0.00 | | | 0.00 | 0.00 | | 0.00 | 0.00 |
| 23.20 | (*E*)-β-caryophyllene | | 9.88 | | 4.90 | | | 19.69 | 8.58 | | | 16.84 | 6.00 | | 32.34 | 16.55 | | 13.20 | 5.81 | | | 15.37 | 9.45 | | | 9.88 | 5.03 | | 9.62 | 3.90 |
| 24.70 | (*E*) -β-farnesene | | 0.00 | | 0.00 | | | 0.14 | 0.14 | | | 0.00 | 0.00 | | 0.00 | 0.00 | | 0.00 | 0.00 | | | 0.00 | 0.00 | | | 0.00 | 0.00 | | 0.00 | 0.00 |
| 24.90 | α-humulene | | 0.00 | | 0.00 | | | 0.71 | 0.44 | | | 0.38 | 0.38 | | 0.87 | 0.87 | | 0.40 | 0.40 | | | 0.52 | 0.52 | | | 0.33 | 0.33 | | 0.27 | 0.27 |
| 25.80 | germacrene D | | 0.00 | | 0.00 | | | 0.00 | 0.00 | | | 0.00 | 0.00 | | 0.00 | 0.00 | | 0.00 | 0.00 | | | 0.00 | 0.00 | | | 0.00 | 0.00 | | 0.00 | 0.00 |
| 26.03 | (*Z*.*E*)-α-farnesene | | 0.70 | | 0.30 | | | 3.27 | 2.01 | | | 1.77 | 1.77 | | 2.10 | 1.62 | | 1.60 | 1.01 | | | 0.28 | 0.28 | | | 3.46 | 3.46 | | 0.71 | 0.71 |
| 26.57 | (*E*.*E*)-α-farnesene | | 19.57 | | 5.54 | | | 82.99 | 49.14 | | | 42.63 | 24.93 | | 30.19 | 9.60 | | 29.96 | 16.20 | | | 10.58 | 6.02 | | | 76.88 | 62.37 | | 23.62 | 15.41 |
| 26.80 | δ-cadinene | | 0.00 | | 0.00 | | | 0.47 | 0.47 | | | 0.47 | 0.47 | | 0.51 | 0.51 | | 1.02 | 0.63 | | | 0.00 | 0.00 | | | 1.38 | 1.05 | | 0.00 | 0.00 |
| 32.56 | nerolidol | | 0.00 | | 0.00 | | | 0.00 | 0.00 | | | 0.00 | 0.00 | | 0.00 | 0.00 | | 0.00 | 0.00 | | | 0.00 | 0.00 | | | 0.08 | 0.08 | | 0.00 | 0.00 |

**Supplemental table 5. Statistical analysis of terpene emission from single *P. trichocarpa* leaves.** Herbivory was imposed by *L. dispar* larvae feeding on either a basal leaf (LPI10), an apical leaf (LPI3) or not at all (control). Volatiles were separately collected from leaves 3 to 10. The terpene emission of the three treatments was compared for each leaf position using a one-way analysis of variance (ANOVA) followed by a factor level reduction. L ratios and p values are shown.

| **LPI** | **linalool** | | **(*E*)-β-Ocimene** | | **DMNT** | | **(*E*)-β-caryophyllene** | | **(*E*,*E*)-α-farnesene** | |
| --- | --- | --- | --- | --- | --- | --- | --- | --- | --- | --- |
| 3  4  5  6  7  8  9  10 | **L ratio**  0.464  1.789  5.789  4.257  0.991  1.549  1.01  1.268 | **p value**  0.508  0.208  **0.0349***  0.0614  0.338  0.237  0.333  0.282* | **L ratio**  486.5  0.493  1.473  0.538  4.491  3.072  1.294  8.433 | **p value**  **<0.0001***  0.497  0.25  0.477  0.0539*  0.105  0.31  **0.0132*** | **L ratio**  9.801  3.975  0.684  1.087  1.124  1.687  3.251  5.33 | **p value**  **0.008**  0.0716  0.426  0.318  0.308  0.218  0.0946  **0.0396*** | **L ratio**  4.284  0.97  5.789  4.015  0.488  0.596  1.703  1.523 | **p value**  0.059*  0.346*  **0.0349***  0.0682*  0.497  0.455  0.215  0.241* | **L ratio**  18.9  0.558  0.015  1.594  0.169  1.29  1.316  15.97 | **p value**  **0.0008***  0.471  0.904*  0.231  0.687  0.278  0.272  **0.00178** |

**Supplemental table 6. Statistical analysis of *TPS* gene expression in individual leaves of *P. trichocarpa*.** Transcript accumulation in herbivore-treated leaves, the closest adjacent leaves and possible vascular connected leaves of the same tree were compared. Trees were either damaged apically (LPI3) or basally (LPI10). Transcript abundance in control trees which received no damage were compared in the same way. A one-way analysis of variance (ANOVA) followed by a factor level reduction was used to test for statistical significance. L ratios and p values are shown.

| **gene** | **treatment** | **transformation** | **pooled leaves** | **L ratio** | **p value** |
| --- | --- | --- | --- | --- | --- |
| *Pttps6* | apical herbivory | log | 3 ; 4+8 | 31.91858 | **<0.0001** |
|  | apical control | - | 3+4+8 | 1.472099 | 0.479 |
|  | basal herbivory | log | 5 ; 9 ; 10 | 27.3099 | **<0.0001** |
|  | basal control | - | 5 ; 9+10 | 11.42989 | **0.0007** |
| *Pttps9* | apical herbivory | - | 3+4+8 | 2.53019 | 0.2822 |
|  | apical control | log | 3 ; 4+8 | 9.0581 | **0.0026** |
|  | basal herbivory | - | 5+9+10 | 0.7776188 | 0.6779 |
|  | basal control | - | 5+9+10 | 2.413139 | 0.2992 |

**Supplemental Table 7.** **Statistical analysis of phytohormone concentrations in single leaves of *P. trichocarpa* in response to *L. dispar* herbivory restricted to a single leaf.**

a) Mixed effects models for each compound (leaf number as fixed effect, plant identity as random effect) were fitted by stepwise exclusion of terms, and were followed by a maximum likelihood ratio test. Models were simplified by factor level reduction (grouped leaves; leaves in different quotation marks are significant different). The results of likelihood ratio tests (= L ratio) and the statistical significance of the explanatory terms (= p values) are listed. b) The influence of the treatment (ha: herbivory on an apical leaf (LPI3); hb: herbivory on a basal leaf (LPI10); ctr: leaves from undamaged trees on phytohormone concentrations was tested with one way ANOVAs. Models were simplified by factor level reduction (grouped treatments; treatments in different quotation marks are significant different). Data of components labelled with * were log transformed for statistical analysis. – indicates that all treatments could be grouped and therefore not different.

|  |  |  |  | |
| --- | --- | --- | --- | --- |
| a) Compound | Treatment | grouped leaves | L ratio | p value |
| SA*  ABA *  JA*  JA-Ile1*  JA-Ile2  OPDA* | ha  ha  ha  ha  ha  ha | "3" "4+5+6+8+10" "7+9"  "3+6" "4+5+7+8+9+10"  "3" "4+5+6+7+8+9+10"  "3" "4+5+7+8+9" "6" "10"  "3" "4+7+10" "5+6+8+9"  "3+4+5" "6+7+8" "9+10" | 35.17591  14.67542  19.87674  51.90763  30.60944  35.32764 | **<0.0001**  **0.0001**  **<0.0001**  **<0.0001**  **<0.0001**  **<0.0001** |
| SA  ABA *  JA*  JA-Ile1*  JA-Ile2*  OPDA* | hb  hb  hb  hb  hb  hb | "3+4+6+7+9" "5+10" "8"  "3" "4+5+10" "6+8+7+9"  "3+4+5+6+7+8+9" "10"  "3+4+5+6+7+8+9" "10"  "3+4+5+6+7+8+9" "10"  "3" "4+5" "6" "7+8+9" "10" | 13.28245  18.06877  22.09746  41.58779  32.52616  44.33695 | **0.0013**  **0.0001**  **<0.0001**  **<0.0001**  **<0.0001**  **<0.0001** |
| SA  ABA *  JA*  JA-Ile1*  JA-Ile2*  OPDA* | ctr  ctr  ctr  ctr  ctr  ctr | "3+4+5" "6+7" "8+9+10"  "3+4+5+6" "7+8+9+10"  "3+4+5+6+9+10" "7+8"  "3+5+6+8+9" "4+7+10"  "3+4+7+8+9+10" "5+6"  "3" "4+5" "6+7" "8+9" "10" | 23.08447  11.155  6.229058  5.955568  6.109897  51.49504 | **<0.0001**  **0.0008**  **0.0126**  **0.0147**  **0.0134**  **<0.0001** |

| b) Compound | Leaf number | grouped treatments | F value | p value |
| --- | --- | --- | --- | --- |
| SA  ABA  JA  JA-Ile1*  JA-Ile2*  OPDA*  SA  ABA  JA  JA-Ile1  JA-Ile2  OPDA  SA  ABA  JA  JA-Ile1  JA-Ile2  OPDA  SA  ABA  JA  JA-Ile1  JA-Ile2  OPDA  SA*  ABA  JA  JA-Ile1  JA-Ile2  OPDA  SA  ABA  JA  JA-Ile1  JA-Ile2  OPDA  SA  ABA  JA  JA-Ile1*  JA-Ile2*  OPDA  SA  ABA  JA  JA-Ile1*  JA-Ile2*  OPDA* | 3  3  3  3  3  3  4  4  4  4  4  4  5  5  5  5  5  5  6  6  6  6  6  6  7  7  7  7  7  7  8  8  8  8  8  8  9  9  9  9  9  9  10  10  10  10  10  10 | “ha” “ctr+hb”  “ha” “ctr+hb”  “ha” “ctr+hb”  “ha” “ctr+hb”  “ha” “ctr+hb”  “ha” “ctr+hb”  “ha+hb” “ctr”  -  -  -  -  -  -  -  -  -  “ha+hb” “ctr”  “ha” “ctr+hb”  -  “ha” “ctr+hb”  “ha+hb” “ctr”  -  -  “ha” “ctr+hb”  -  “ha” “ctr+hb”  -  -  -  -  “ha” “ctr+hb”  “ha” “ctr+hb”  -  -  -  -  -  -  -  -  “hb” “ctr+ha”  -  -  -  “hb” “ctr+ha”  “hb” “ctr+ha”  “hb” “ctr+ha”  - | 6.687  3.428  7.104  34.61  40.88  9.639  3.421  0.058  1.88  0.069  0.698  2.46  0.83  1.527  2.839  2.191  3.663  8.383  2.726  10.32  4.334  1.555  2.165  6.47  2.933  3.629  0.243  0.952  0.7  0.843  7.144  5.25  1.12  1.983  0.723  1.542  0.534  2.219  2.429  2.258  4.933  2.609  2.772  0.829  8.153  92.6  24.53  2.186 | **0.0226**  0.0869  **0.01943**  **<0.0001**  **<0.0001**  **0.00837**  0.0914  0.815  0.198  0.798  0.421  0.145  0.379  0.238  0.116  0.163  0.0779  **0.0125**  0.123  **0.0068**  0.0577  0.234  0.165  **0.0245**  0.11  0.0791  0.63  0.347  0.418  0.375  **0.0192**  **0.0393**  0.309  0.182  0.41  0.236  0.479  0.162  0.145  0.159  **0.0463**  0.132  0.124  0.382  **0.0156**  **<0.0001**  **0.0004**  0.167 |

**Supplemental table 8. Terpenes present in the herbivore-induced volatile blend of *P. trichocarpa* leaves and terpene synthases able to mediate their formation.**

| **terpene** | **PtTPS possibly involved in production** |
| --- | --- |
| **Monoterpenes** |  |
| α-pinene | PtTPS13 |
| myrcene | PtTPS13 |
| limonene | - |
| 1,8-cineole | PtTPS13 |
| (*Z*)-β-ocimene | PtTPS6 |
| (*E*)-β-ocimene | PtTPS6 |
| alloocimene | PtTPS6 |
| linalool | PtTPS3 / PtTPS12 |
| (*E*)-epoxy-ocimene | PtTPS6 |
| citronellol | - |
|  |  |
| **Homoterpenes** |  |
| DMNT  TMTT | PtTPS4 / PtTPS15  PtTPS10 |
| **Sesquiterpenes** |  |
| α-cubebene | PtTPS1 |
| α-copaene | PtTPS1 |
| (*E*)-β-caryophyllene | PtTPS9 (PtTPS7) |
| (*E*)-β-farnesene | PtTPS4 / PtTPS2 |
| α-humulene | PtTPS9 (PtTPS7) |
| germacrene D | PtTPS1 (PtTPS7 / PtTPS14) |
| (*Z*,*E*)-α-farnesene | PtTPS2 |
| (*E*,*E*)-α-farnesene | PtTPS2 |
| δ-cadinene | PtTPS1 |
| nerolidol | PtTPS15 |

## Supplemental table 9. Volatiles emitted from excised *P. trichocarpa* leaves treated with jasmonic acid (ng g^-1^ fresh weight h^-1^).

Leaves were kept in water (ctr) or jasmonic acid (JA, 250 µM) for 18h. Volatiles were collected and analyzed using GC-MS and GC-FID. Means and standard errors (SE) are shown (n = 4). * indicates significant differences (p < 0.05) between the treatments.

| Volatiles | ctr | SE | JA | SE |
| --- | --- | --- | --- | --- |
| **terpenoids** |  |  |  |  |
| myrcene * | 0.98 | 0.34 | 4.88 | 1.04 |
| limonene * | 0.42 | 0.42 | 2.19 | 0.48 |
| (*Z*)-β-ocimene * | 2.53 | 0.91 | 23.15 | 2.40 |
| (*E*)-β-ocimene * | 39.71 | 11.89 | 400.01 | 45.00 |
| linalool | 2.01 | 1.20 | 9.24 | 4.09 |
| DMNT | 16.52 | 4.28 | 16.16 | 1.30 |
| (*E*)-epoxy-ocimene * | 1.24 | 0.49 | 22.56 | 5.57 |
| α-cubebene * | 0.00 | 0.00 | 1.78 | 0.30 |
| α-copaene * | 0.00 | 0.00 | 1.65 | 0.41 |
| (*E*)-β-caryophyllene | 82.27 | 27.37 | 222.97 | 65.18 |
| α-humulene | 5.09 | 1.57 | 13.41 | 3.22 |
| germacrene D * | 5.03 | 3.01 | 27.20 | 3.50 |
| (*Z*,*E*)-α-farnesene * | 5.95 | 1.68 | 15.23 | 2.26 |
| (*E*,*E*)-α-farnesene * | 218.08 | 51.64 | 594.73 | 106.89 |
| TMTT | 6.67 | 1.10 | 6.45 | 0.47 |
| **other volatiles** |  |  |  |  |
| (*E*)-3/2-methylbutyraldoxime | 2.42 | 0.90 | 21.12 | 10.77 |
| (*Z*)-2-methylbutyraldoxime | 0.00 | 0.00 | 7.31 | 3.66 |
| (*Z*)-3-methylbutyraldoxime | 0.00 | 0.00 | 8.28 | 8.28 |
| (*Z*)-3-hexenol | 44.56 | 14.40 | 170.30 | 152.81 |
| 1-hexanol | 9.43 | 3.30 | 310.58 | 306.91 |
| isoamylacetat | 0.89 | 0.31 | 1.20 | 0.47 |
| benzaldehyde | 4.27 | 1.71 | 34.09 | 22.33 |
| (*Z*)-3-hexenyl acetate | 42.35 | 18.21 | 184.61 | 170.94 |
| N-hexyl acetate | 4.99 | 1.75 | 109.85 | 105.30 |
| (*E*)-2-hexenyl acetate | 3.25 | 1.27 | 28.09 | 23.05 |
| benzyl alcohol | 2.68 | 0.92 | 8.86 | 4.04 |
| salicylaldehyde | 0.00 | 0.00 | 2.71 | 2.71 |
| nonanal | 4.10 | 0.98 | 5.05 | 1.84 |
| phenylethyl alcohol * | 0.00 | 0.00 | 14.09 | 6.54 |
| benzyl cyanide * | 3.17 | 0.83 | 65.48 | 19.59 |
| benzoic acid | 11.00 | 6.65 | 2.90 | 1.11 |
| methyl salicylate | 2.94 | 1.16 | 0.69 | 0.69 |
| (*E/Z*)-phenylacetaldoxime | 0.00 | 0.00 | 3.29 | 3.08 |
| phenylethyl acetate | 0.00 | 0.00 | 1.76 | 1.10 |
| indol * | 2.26 | 0.89 | 42.41 | 11.03 |
| 2-phenylnitroethane | 0.00 | 0.00 | 2.12 | 1.24 |

**Jasmonic acid (JA) induction experiment**

To investigate whether JA is able to induce volatile emission from *P. trichocarpa* leaves, excised leaves were placed into solutions containing tap water or tap water + JA (250 µM). After an 18 h overnight incubation, volatiles released from these leaves were collected and analyzed using gas chromatography-mass spectrometry (DB-WAX column) as described in Irmisch et al. (2013).

**Supplemental table 10. Oligonucleotides used for isolation and qRT-PCR analysis of *TPS* genes.**

| Name | Sequence | Usage |
| --- | --- | --- |
| PtTPS5-fwd | ATGGCCCTTCAAGAATCAACCC | isolation |
| PtTPS5-rev | TCATGCTACAACTGGAAGGGGA | isolation |
| PtTPS6-fwd | ATGCCGAGGCATCCTCTGCC | isolation |
| PtTPS6-rev | TTAATGAAATGAAATGGGTTCAATTA | isolation |
| PtTPS7-fwd | ATGTCTACCCAAGTCTCCCAAG | isolation |
| PtTPS7-rev | TTATATTGGCACTGAATTTATAAG | isolation |
| PtTPS8-fwd | ATGGCATTGCAGACAGATTTACCAGC | isolation |
| PtTPS8-rev | TTAAAGGGATATTTTCTCCACGAACAATAAAC | isolation |
| PtTPS9-fwd | ATGTCCTCTCATGTTTCAGCTGC | isolation |
| PtTPS9-rev | CTATGAAGCTAATAAGCTCGGAG | isolation |
| PtTPS10-fwd | ATGGAATTTTCAAAATCTTTCAATATTC | isolation |
| PtTPS10-rev | TCACATGAAACAGAATTTTAACTTCGGTG | isolation |
| PtTPS11-fwd | ATGGAAAACACCAATCAACAAAAC | isolation |
| PtTPS11-rev | TTATTCATACAGAGGCACTTGC | isolation |
| PtTPS12-fwd | ATGGCTCCTACCCACTTGGATTCATC | isolation |
| PtTPS12-rev | TTATAAAGGCTTAATAAGTAAGGACATCACCC | isolation |
| PtTPS13-fwd | ATGGCTCTTACCCACTTTGCTTCATC | isolation |
| PtTPS13-rev | TTATAAAGGCTTAATAAGTAAGGATTTCACCC | isolation |
| PtTPS14-fwd | ATGGAAACCATAACAACTCTTCGTTTAACAG | isolation |
| PtTPS14-rev | TTATTCACACATAGGCACTTGCTCAAC | isolation |
| PtTPS15-fwd | ATGGCATTTCCCATCAATATTGATGGCAAC | isolation |
| PtTPS15-rev | CTAGAAAGGTTTGCTTTCTAAATGAGCAGC | isolation |
| PtTPS5-QRTfwd | GCACTGGAGCTGTTCTAAAG | qRT-PCR |
| PtTPS5-QRTrev | GGGTAGCTTTGTAGCAAAGTC | qRT-PCR |
| PtTPS6-QRTfwd | CGATCGGTATCAGGAACTGTC | qRT-PCR |
| PtTPS6-QRTrev | GCTATCTCTGCTGATGAAGTG | qRT-PCR |
| PtTPS7-QRTfwd | GAGAGCAGGCGCTACATTTC | qRT-PCR |
| PtTPS7-QRTrev | CCACCTTGTAATTTCTGCAAGC | qRT-PCR |
| PtTPS9-QRTfwd | TAGTGGTGGCGCCTATGTC | qRT-PCR |
| PtTPS9-QRTrev | GTGAACCCTGAGGTGTATAGC | qRT-PCR |
| PtTPS10-QRTfwd | GAAGCATTGCCTGCTCTATATAA | qRT-PCR |
| PtTPS10-QRTrev | GTTTCCAGTAGCCATGAAAGC | qRT-PCR |
| PtTPS11/14-QRTfwd | GCAGACCATATTAGGAATGCTT | qRT-PCR |
| PtTPS11/14-QRTrev | TTGGCAAACTTGAGTAGAGTG | qRT-PCR |
| PtTPS12-QRTfwd | GATTTCAGTTGGAGCACCATTGGC | qRT-PCR |
| PnTPS12-QRTrev | CTCTTTTTATTTCATCCGAGGAGG | qRT-PCR |
| PnTPS13-QRTfwd | GGTGGAGAATTTCTTATGGACTGTG | qRT-PCR |
| PtTPS13-QRTrev | GGAAGCCGATCCATGTAATTTAG | qRT-PCR |
| PtTPS15-QRTfwd | ATGACGTAAAGGGTTTGATGG | qRT-PCR |
| PtTPS15-QRTrev | GCTCTTGTGATAAGGATGCTTC | qRT-PCR |
